# Discovery of Potent Tetrazole Free Fatty Acid Receptor 2 Antagonists

**DOI:** 10.1021/acs.jmedchem.2c01935

**Published:** 2023-05-02

**Authors:** Alice Valentini, Katrine Schultz-Knudsen, Anders Højgaard Hansen, Argyro Tsakoumagkou, Laura Jenkins, Henriette B. Christensen, Asmita Manandhar, Graeme Milligan, Trond Ulven, Elisabeth Rexen Ulven

**Affiliations:** † Department of Drug Design and Pharmacology, University of Copenhagen, Universitetsparken 2, DK-2100 Copenhagen, Denmark; ‡ Centre for Translational Pharmacology, School of Molecular Biosciences, College of Medical, Veterinary and Life Sciences, University of Glasgow, Glasgow G12 8QQ, Scotland, United Kingdom; § Department of Physics, Chemistry and Pharmacy, University of Southern Denmark, Campusvej 55, DK-5230 Odense M, Denmark

## Abstract

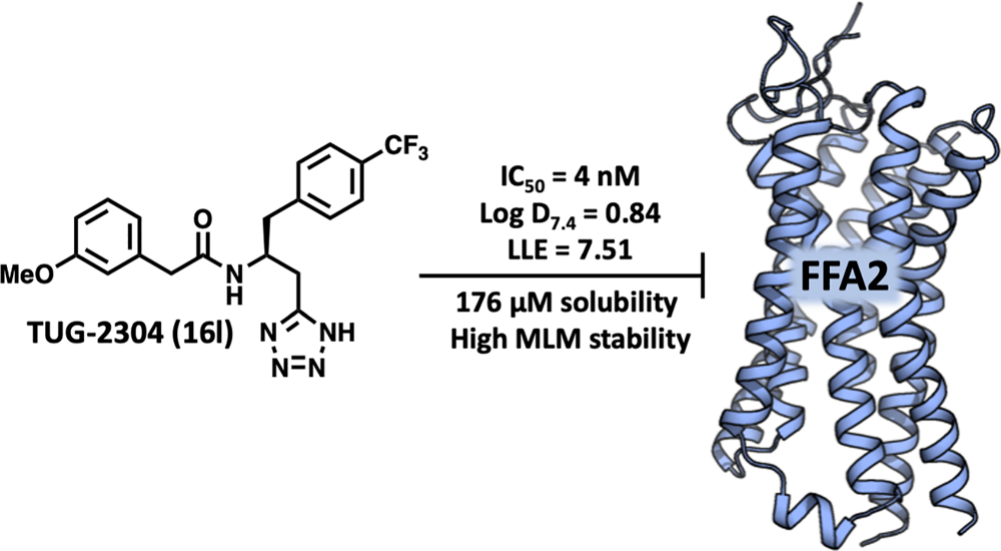

The free fatty acid receptor 2 (FFA2), also known as GPR43, mediates effects of short-chain fatty acids and has attracted interest as a potential target for treatment of various metabolic and inflammatory diseases. Herein, we report the results from bioisosteric replacement of the carboxylic acid group of the established FFA2 antagonist CATPB and SAR investigations around these compounds, leading to the discovery of the first high-potency FFA2 antagonists, with the preferred compound TUG-2304 (**16l**) featuring IC_50_ values of 3–4 nM in both cAMP and GTPγS assays, favorable physicochemical and pharmacokinetic properties, and the ability to completely inhibit propionate-induced neutrophil migration and respiratory burst.

## Introduction

Short-chain fatty acids (SCFAs), especially acetate, propionate, and butyrate, are the main metabolites of the gut microbiota, produced in high concentrations by fermentation of dietary fiber, and responsible for regulation of a variety of physiological effects.^1,2^ These include effects on metabolism, the immune system, and the nervous system that are at least partly mediated by free fatty acid receptor 2 (FFA2, also known as GPR43) and the closely related FFA3 (GPR41), both G protein-coupled receptors.^3−8^ The receptors are expressed together or selectively in various tissues such as the small intestine and colon,^9^ pancreas,^10^ adipose tissue, lungs, and ganglia.^11^ Both FFA2 and FFA3 have been linked to effects on regulation of body weight and glucose,^12,13^ and the receptors are suggested as therapeutic targets for type 2 diabetes,^14^ asthma,^15−17^ non-alcoholic steatohepatitis,^18^ and chronic kidney disease.^19^ A study showed that double FFA2/FFA3 knockout mice had improved insulin secretion, suggesting that inhibition of the receptors may represent a therapy of hyperglycemia.^10^ This is supported by the observation that FFA2 agonists inhibit glucose-stimulated insulin secretion.^20^ FFA2 is expressed on immune cells including neutrophils, dendritic cells, and macrophages^21,22^ and is proposed as a target for inflammatory bowel disease,^23^ sepsis,^24^ and rheumatoid arthritis.^25^ FFA2 was also recently found to assist influenza A infection^26^ and has appeared as a new target for treatment of lung infection.^26−29^

**Chart 1:**
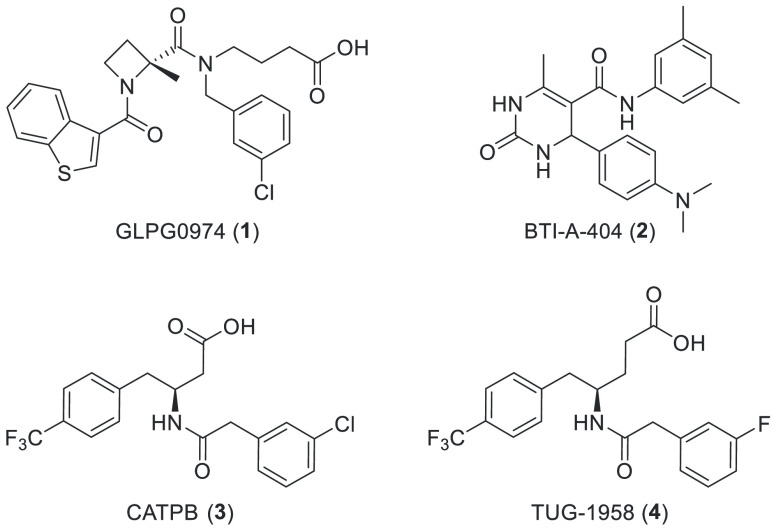
FFA2 Antagonists

Although numerous studies suggest therapeutic potential for FFA2 and FFA3 in various diseases, results in several areas conflict regarding the need for activation or inhibition, and additional validating studies are required. Many studies have relied on transgenic mice, but issues related to reported cross-effect on expression in knockouts have suggested that results should be interpreted with care,^30^ and the lack of high-quality tool compounds has been a limiting factor. For FFA3, besides the SCFAs, the only options are more selective but low-potency small carboxylic acids^31^ and a series of moderate-potency allosteric agonists.^32,33^ The situation is somewhat better for FFA2 with several orthosteric, allosteric, and biased agonists available.^34−37^ In terms of FFA2 antagonists, the Galapagos compound GLPG0974 (**1**, Chart 1) is so far the only FFA2 antagonist that has been in clinical trials but failed to reach the endpoint for treatment of ulcerative colitis despite observed inhibition of neutrophil influx.^23,38^ BTI-A-404 (**2**) is an inverse agonist, reportedly with an IC_50_ of only ∼10 μM, but still capable of enhancing GLP-1 secretion from a colon cell line.^39^ CATPB (**3**) is an FFA2 antagonist with inverse agonist properties discovered by Euroscreen.^40,41^ The potency and affinity of **3** are similar to or slightly better than **1**, but its kinetic properties differ with higher on- and off-rates.^42^

We recently reported an SAR study around **3** and the discovery that extension of the carboxylate chain by one methylene unit increased potency, resulting in the identification of TUG-1958 (**4**) as the most potent FFA2 antagonist reported hitherto.^43^ Herein, we report the further investigations around this series with focus on bioisosteric replacement of the carboxylic acid group with a tetrazole and the finding that this provides FFA2 antagonists with favorable properties and significantly further improved potency to the low nanomolar range.

## Synthesis

**Scheme 1:**
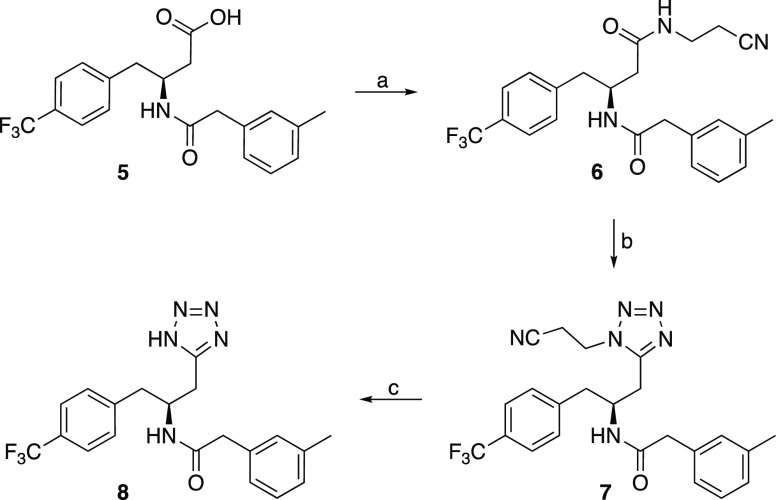
Initial Tetrazole Synthesis Reagents and conditions: (a) 3-aminopropionitrile, HOBt, EDC^.^HCl, DIPEA, DMF, rt, overnight, 62%; (b) PPh_3_, MeCN, DIAD, TMSN_3_, 0 °C to 50 °C, overnight, 16%; (c) LiOH, H_2_O, THF, rt, 2 h, 61%.

**Scheme 2:**
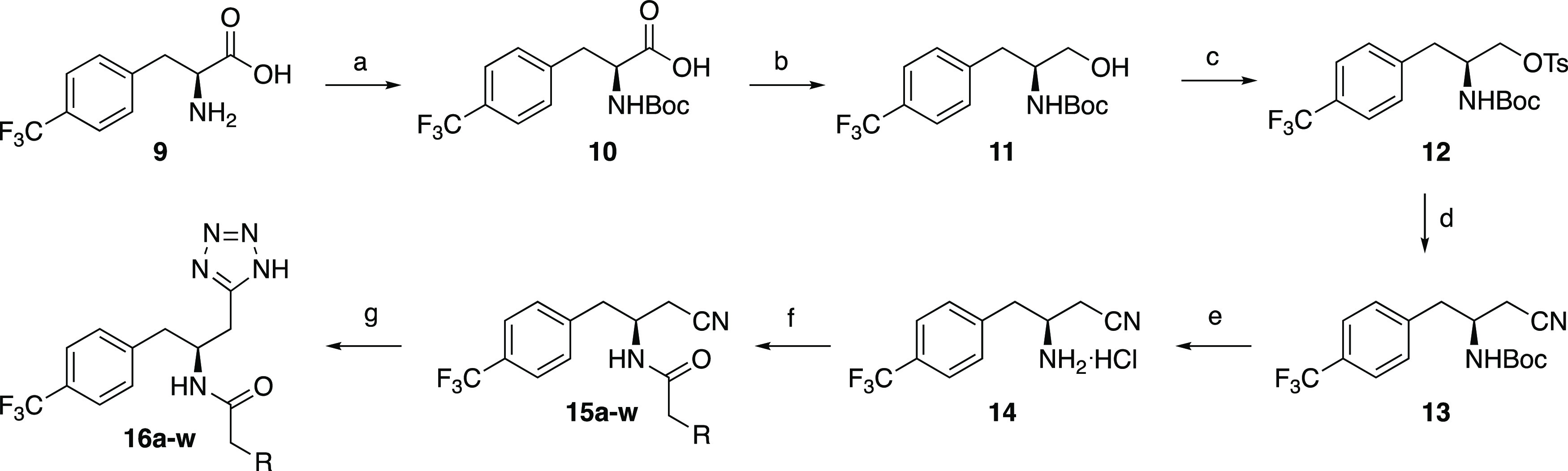
Optimized Synthetic Route Reagents and conditions: (a) Boc_2_O, Et_3_N, MeOH/H_2_O, 55 °C, overnight, 97%; (b) (i) IBCF, NMM, THF, −15 °C, 1.5 h, (ii) NaBH_4_, −15 °C, 1 h, 64%; (c) TsCl, Et_3_N, DMAP, DCM, 0 °C → rt, 3.5 h, 85%; (d) NaCN, DMF, 0 °C → 60 °C, 5 h, 82%; (e) 4 M HCl in dioxane, DCM, rt, overnight, 100%; (f) PyBOP, RCOOH, DIPEA, DMF, 0 °C → rt, overnight, 29–86%; (g) NaN_3_, Et_3_N^.^HCl, toluene, reflux, 16–48 h, 5–95%.

The first tetrazole (**8**) was synthesized from **5**, an equipotent methyl analogue of **3**,^43^ by coupling with 3-aminopropionitrile to form **6** followed by DIAD-promoted reaction with TMS-azide to **7** and finally basic deprotection to give the tetrazole **8** (Scheme 1).^44^ However, since the overall yield was low and the laborious final introduction of the tetrazole was not optimal for SAR exploration, we set out to identify a more efficient route. We chose to start from the phenylalanine derivative **9** since this substrate would introduce the correct stereochemistry. Boc-protection (**10**),^45^ reduction of the carboxylic acid to the corresponding alcohol **11**, tosylation (**12**), substitution by cyanide (**13**), and deprotection provided the aminonitrile intermediate **14** (Scheme 2).^46,47^ PyBOP-promoted amide coupling with **14** allowed diversifications before the tetrazole was installed by reaction with sodium azide in the final step.

**Scheme 3:**
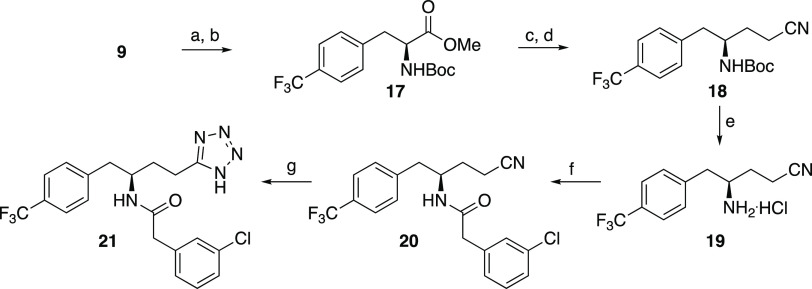
Synthesis of Homologated Tetrazoles Reagents and conditions: (a) SOCl_2_, MeOH, 0 °C → rt, overnight, 81%; (b) Boc_2_O, DIPEA, DCM, 0 °C → rt, overnight, 89%, (c) (i) DIBALH, DCM, −78 °C, 80 min; (ii) Ph_3_PCH_2_CN^.^Br, KOtBu, rt, overnight; (d) H_2_, Pd/C, EtOH, rt, overnight, 39% over two steps; (e) 4 M HCl in dioxane, 0 °C → rt, 2 h, 99%; (f) 3-chlorophenylacetic acid, PyBOP, DIPEA, DMF, 0 °C → rt, overnight, 64%; (g) NaN_3_, ZnBr_2_, H_2_O, *i*PrOH, 130 °C (μν), 17%.

The homologated tetrazole **21** was also synthesized from **9** by conversion to the corresponding methyl ester and Boc-protected **17** followed by a DIBALH reduction to the aldehyde, a Wittig reaction on the crude product, and hydrogenation to give **18** (Scheme 3). Deprotection to **19**, amide coupling to **20**, and tetrazole formation by using sodium azide and zinc bromide under microwave irradiation provided **21**.

## Results and Discussion

The activity of the compounds on FFA2 was evaluated in two different functional antagonist assays, both using propionate at EC_80_ as the agonist. The GTPγS assay reflects receptor activation by directly assessing G protein recruitment, whereas the cAMP assay, performed in whole cells, reflects a physiologically relevant downstream second messenger.

To our excitement, the initial tetrazole **8** showed more than 10-fold increased potency over CATPB (**3**) and a 4-fold increase over the recently published FFA2 antagonist TUG-1958 (**4**),^43^ thus representing the most potent FFA2 antagonist identified so far by a good margin (Table 1). The high potency was confirmed in the cAMP assay; thus, **8** was found to inhibit the effect of propionate with an IC_50_ of 6–9 nM.

**Table 1:**
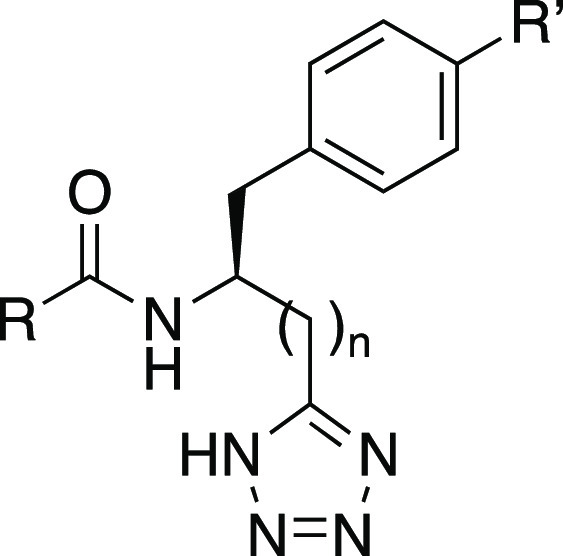

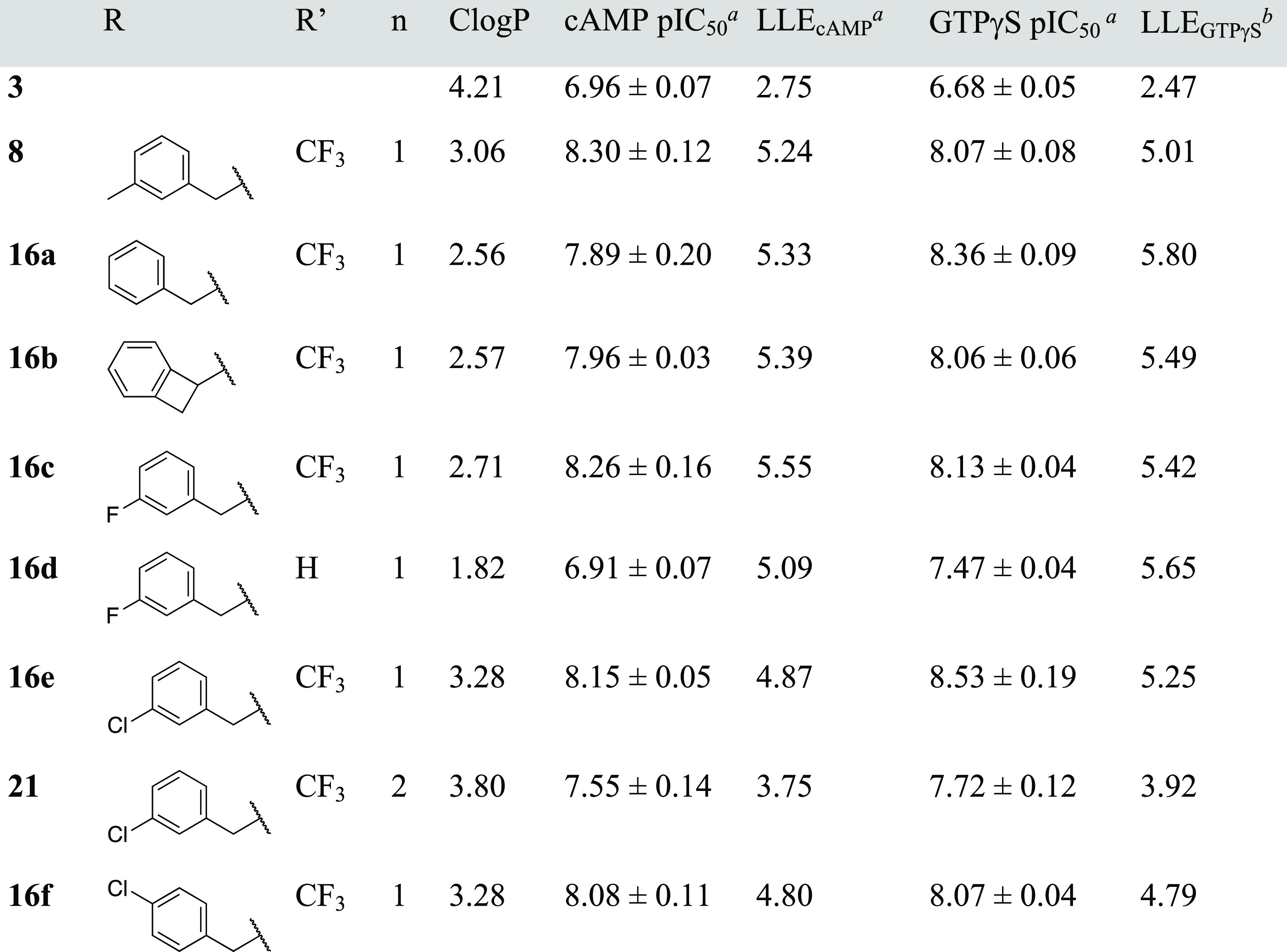
SAR Exploration of the Acetamide Aryl on hFFA2

a Values are mean ± SEM from three or more independent experiments, each performed in duplicate or triplicate.

b LLE_cAMP_ = pIC_50, cAMP_ – ClogP.

c LLE_GTPγS_ = pIC_50, GTPγS_ – ClogP.

Our previous work on homologated CATPB analogues had shown that modification of the carboxylic acid part of this compound class can affect the SAR in other parts of the molecule.^43^ Taking advantage of our improved synthetic route (Scheme 2), we focused on exploration of the phenylacetamide group. We paid special attention to ClogP since high lipophilicity has been an issue with previous FFA2 ligands, as with free fatty acid receptor ligands in general.^43,48^

The unsubstituted phenylacetamide **16a** was only slightly less potent than *meta*-methyl **8** in the cAMP assay and tended toward increased potency in the GTPγS assay, indicating that the methyl is not essential for activity. Interestingly, the constrained benzocyclobutane **16b** also displayed similar potency.

Introducing the smaller and more electronegative *meta*-fluoro **16c**, which was found to be preferred on the homologated antagonist series,^43^ did not affect the potency. In contrast, removing the trifluoromethyl from the other benzene ring (**16d**) reduced the potency by a log unit in the cAMP assay and somewhat less in the GTPγS assay, clearly showing that this substituent remains important for activity also in the tetrazole series. Still, the lipophilic nature of CF_3_ makes the LLE similar for the compounds.

Exchanging the methyl of **8** to the size-similar but electronegative chloro (**16e**) gave essentially an equipotent compound. Next, inspired by the increased potency of **4** vs **3** (Chart 1), we elongated the tetrazole linker in **21**; however, in this case the potency decreased 5–6-fold in the assays. Presuming a similar binding mode, this could be due to the somewhat larger size of the tetrazole compared to the carboxylic acid. In the further exploration of the phenylacetamide, a *para*-chloro substituent (**16f**) showed preserved (cAMP) or slightly reduced (GTPγS) potency. Synthesis of the analogue with an *ortho*-chloro substituent was also attempted but the tetrazole synthesis failed for this compound. Due to the pronounced reduction in potency for *ortho*-substituted phenylacetamides with carboxylate scaffolds,^43^ this was not explored further.

Continuing our exploration of the *meta*-substituted phenylacetamides, the bromo (**16g**) or iodo (**16h**) did not affect the potency but reduced LLE by increasing ClogP (Table 2). Introduction of the larger and more electronegative trifluoromethyl (**16i**) was tolerated but with a somewhat reduced potency. Exploring less lipophilic substituents, we first introduced a nitrile (**16j**), which lowers lipophilicity by one log unit, but this led to a reduction in potency of at least two log units, while a tetrazole (**16k**), obtained as a byproduct in the synthesis of **16j**, was completely inactive. In contrast, exploration of alkoxy substituents revealed that the methoxy (**16l**) improved the potency in both assays and provided the most potent compound thus far. The larger ethoxy (**16m**) and trifluoromethoxy (**16n**) resulted in comparably reduced potency. This demonstrates that the lipophilicity can be lowered by adding more polar substituents while preserving or increasing potency and furthermore suggests that the binding pocket accommodating the *meta*-position substituents has a limited size.

**Table 2:**
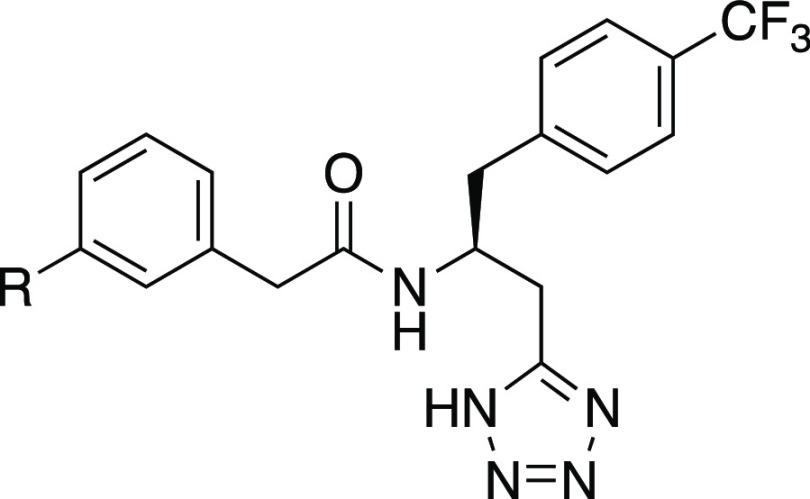

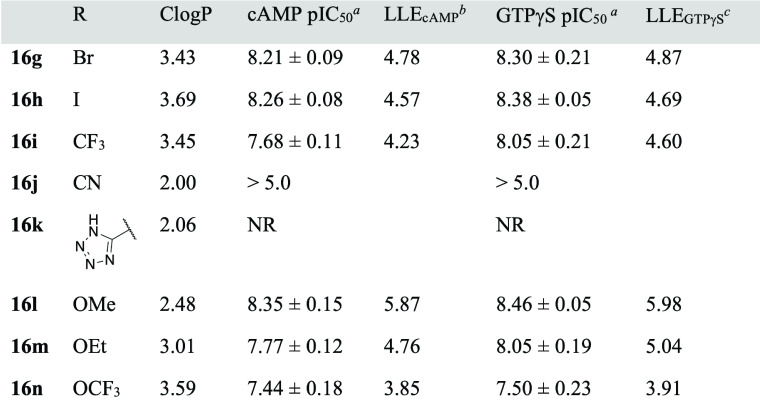
Exploration of *meta*-Substituents on the Phenylacetamide Part

a Values are mean ± SEM from three or more independent experiments, each performed in duplicate or triplicate. NR, no response.

b LLE_cAMP_ = pIC_50, cAMP_ – ClogP.

c LLE_GTPγS_ = pIC_50, GTPγS_ – ClogP.

Next, we explored aliphatic groups with similar ClogP values to avoid effects purely based on lipophilicity (Table 3). The cyclopentylacetamide (**16o**) showed low potency, while the longer and more flexible hexanamide (**16p**) displayed comparably improved activity that was further improved by reintroducing the phenyl as phenylpropanamide (**16q**) but still clearly inferior to the phenylacetamide series.

**Table 3:**
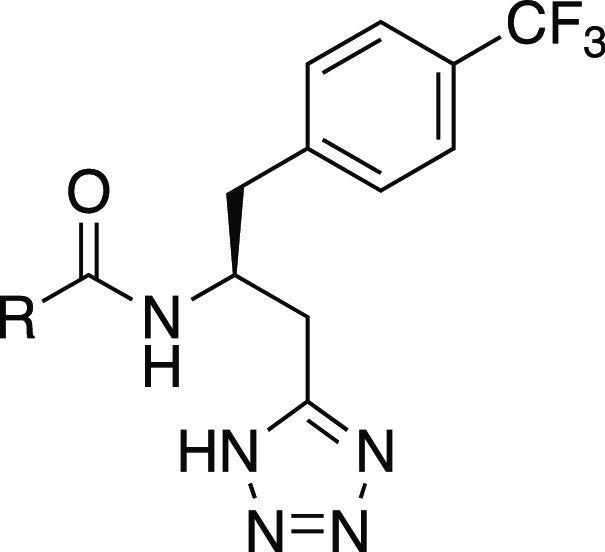

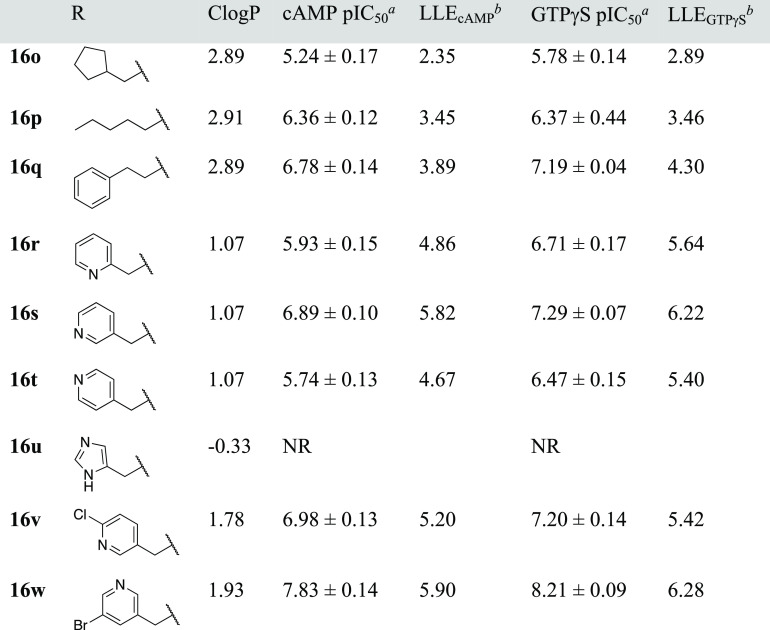
Exploration of Aliphatic and Heteroaromatic Variations of the Phenylacetamide

a Values are mean ± SEM from three or more independent experiments, each performed in duplicate or triplicate. NR, no response.

b LLE_cAMP_ = pIC_50, cAMP_ – ClogP.

c LLE_GTPγS_ = pIC_50, GTPγS_ – ClogP.

In a further attempt to reduce lipophilicity, we explored a nitrogen walk in the phenylacetamide. All analogues showed reduced potency, as expected from the reduced lipophilicity, but the 3-pyridyl (**16s**) was clearly favored with a moderate 7-fold decrease in both assays, while the 2-pyridyl (**16r**) and the 4-pyridyl (**16t**) showed pronouncedly decreased potencies in both assays compared to **16a**, with the strongest reduction in the cAMP assay. Introduction of the even more polar imidazole (**16u**) resulted in a completely inactive compound.

Despite the reduced potency of the 3-pyridyl **16s** compared to the phenyl analogue **16a**, the greater reduction in lipophilicity resulted in the highest LLE observed thus far. We hypothesized that adding a small substituent to the pyridine ring could boost the potency as seen with the phenyl analogues while keeping a low lipophilicity. Thus, based on accessible building blocks, we decided to investigate two compounds. Introducing a *para*-chloro (**16v**) was tolerated but did not lead to an increase in potency, whereas the *meta*-bromo-substituted pyridine analogue (**16w**) led to a 9-fold increase in potency and the highest ClogP-based LLE value of all compounds.

The most promising compounds were selected for further characterization, including kinetic aqueous solubility, log D_7.4_, and chemical and microsomal stability. The log D_7.4_ values were found to correlate well with ClogP values (*r*^2^ = 0.84), as also previously observed for another series,^49^ validating the ClogP-based LLE values above. The methyl analogue **8** was found to have a good solubility and appropriate lipophilicity, whereas the corresponding chloro analogue **16e** showed somewhat reduced solubility, further reduced in the bromo (**16g**) and iodo (**16h**) analogues (Table 4). The bromopyridine **16w** had the highest solubility and lowest experimental log D_7.4_, whereas methoxyphenyl **16l** also exhibited good solubility and had the highest experimental LLE of 7.5 (vs LLE 7.1 for **16w**). Both compounds showed full chemical stability with >95% remaining after 10 days in PBS_7.4_ at 37 °C and low in vitro clearance as evaluated in mouse liver microsomes (Figure S1) and were also devoid of activity on the closely related receptor FFA3 at 10 μM. The overall preferred compound **16l** was found to have satisfactory pharmacokinetic properties in mice (Table 5).

**Figure 1:**
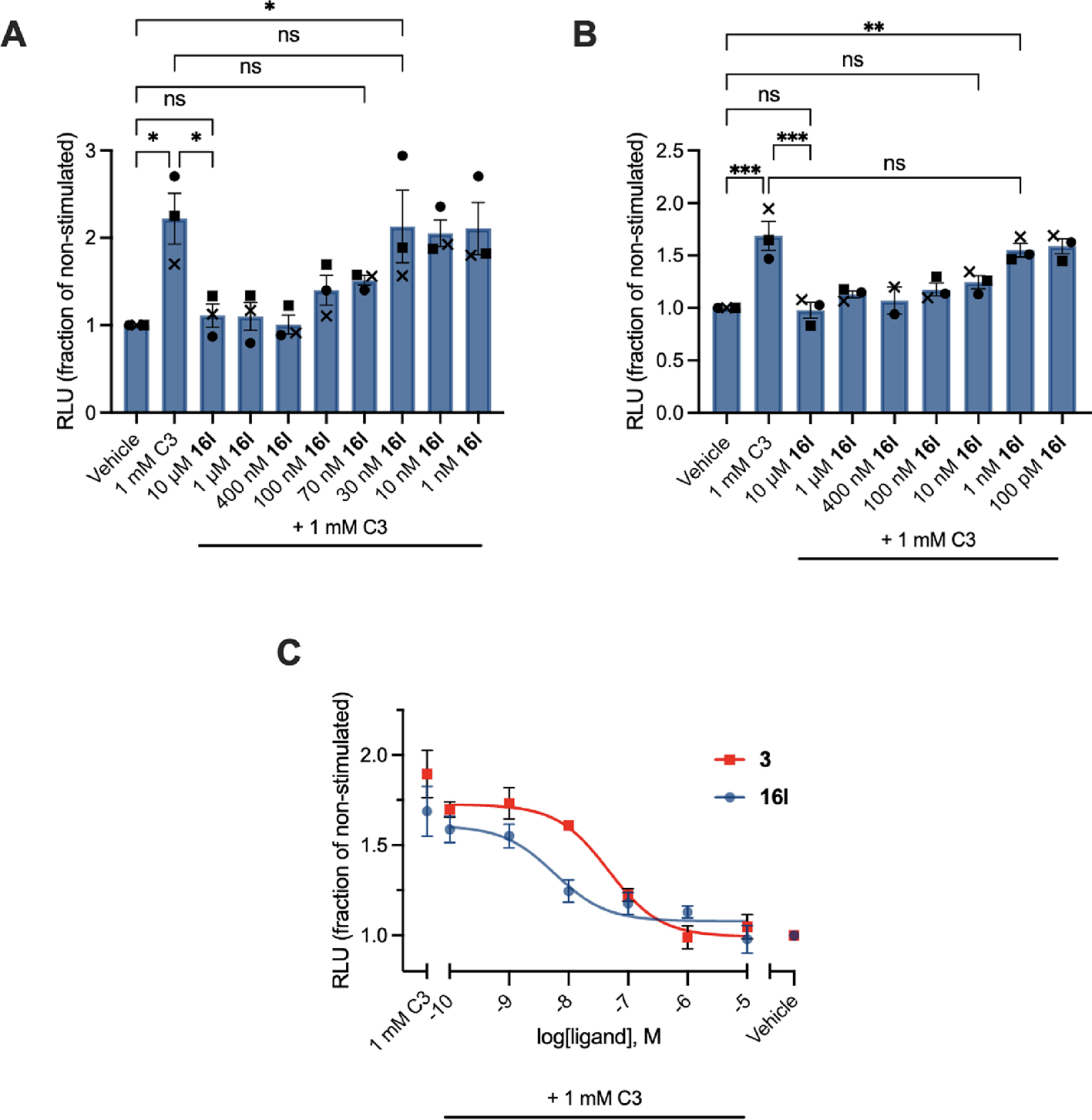
Compound **16l** inhibited propionate (C3, 1 mM)-induced (A) migration and (B) respiratory burst of human neutrophils. (C) Concentration–response curves of **3** (pIC_50_ = 7.33) and **16l** (pIC_50_ = 8.24) on C3-induced (1 mM) respiratory burst. The solid circle, square, and cross marks represent the mean of each independent experiment. Bars represent mean ± SEM from three biologically independent experiments, each performed in triplicate. *, *p* < 0.05; **, *p* < 0.005; ***, *p* < 0.001; ns, not significant.

**Table 4:** Physicochemical Characterization of Selected Compounds

	solubility (μM)^a^	logD_7.4_^b^	LLE_cAMP-logD_^c^	chemical stability^d^	microsomal stability^e^
**3**	166	1.39 ± 0.01	5.57	90%	91%
**8**	163	1.26 ± 0.07	7.04		89%
**16e**	138	1.80 ± 0.09	6.35		92%
**16g**	121				
**16h**	81				
**16l**	176	0.84 ± 0.01	7.51	>95%	93%
**16w**	183	0.71 ± 0.06	7.12	>95%	92%

a Kinetic solubility in PBS at pH 7.4 and 25 °C.

b Mean of two independent experiments.

c LLE_cAMP_ = pIC_50, cAMP_ – logD_7.4_.

d PBS_7.4_ at 37 °C, 10 days.

e % recovered after 60 min.

**Table 5:** Pharmacokinetic Properties of **16l** in Mice

iv admin (5 mg/kg)	
*t*_1/2_	24 min
AUC_0–∞_	441,000 ng min mL^–1^
*V*_d_	400 mL kg^–1^
CL_total_	11 mL min^–1^ kg^–1^
po admin (10 mg/kg)	
*t*_max_	30 min
*C*_max_	6240 ng mL^–1^
AUC_0–∞_	389,000 ng min mL^–1^
*F*%	44%

Inhibition of neutrophil activation and migration is a suggested mechanism by which FFA2 antagonists can counteract inflammatory diseases.^50−54^ Thus, we evaluated the ability of **16l** to inhibit propionate-induced migration of human neutrophils and observed that the compound was able to fully inhibit migration (Figure 1A). We also investigated the activity of **16l** on propionate-induced respiratory burst by its effect on luminol-amplified chemiluminescence and found full inhibition of the propionate-induced effect (Figure 1B). Importantly, **16l** did not exhibit any effect on neither neutrophil migration nor respiratory burst induced by fMLP, indicating that the effect is specific for C3-indued neutrophil activation and mediated by FFA2 (Supporting Figure S2). Dose–response curves from the neutrophil respiratory burst assay revealed 8-fold higher potency of **16l** compared to **3** (Figure 1C).

In conclusion, replacement of the carboxylic acid head group of the established FFA2 antagonist CATPB (**3**) by tetrazole results in a novel antagonist series with more than an order of magnitude increased potency and the first high-potency FFA2 antagonists. The preferred compound **16l** inhibited propionate at FFA2 with IC_50_ 4 nM in both the cAMP and GTPγS assays and showed low lipophilicity, favorable aqueous solubility, high chemical and microsomal stability, favorable pharmacokinetic properties in mice, and >10,000-fold selectivity over FFA3. Finally, physiologically relevant activity of **16l** was demonstrated by complete inhibition of propionate-induced neutrophil activation and respiratory burst.

## Experimental Section

All reagents were of commercial grade and used as received without further purification, unless otherwise stated. Anhydrous reactions were carried out under an argon atmosphere in flame-dried glassware. DCM, THF, and DMF were dried with a Waters SG solvent purification system. Water was deionized, while water for HPLC was deionized and filtered (MilliQ). DIPEA was dried over 3 Å sieves. Pre-coated TLC plates with silica gel 60 F254 (Merck) were used. Purification of compounds was performed using silica gel 60 (0.040–0.063 mm, Merck) manually or by automated flash chromatography on a Büchi Reveleris X2. Preparative HPLC was performed on a Thermo Scientific Ultimate HPLC system using a Gemini-NX C18 column (Phenomenex); mobile phase A: 0.1% v/v TFA, 100% H_2_O (v/v); mobile phase B: 0.1% v/v TFA, 10% H_2_O, 90% MeCN (v/v); gradient elution: 20–100% B over 15 min or 0–100% B over 15 min. NMR spectra were recorded on 400 or 600 MHz Bruker instruments and calibrated after residual solvent peaks. Mass spectrometry (MS) was performed on a Waters Aquity UPLC instrument with an Aquity QDa detector or on an Agilent 6130 Mass Spectrometer instrument using electron spray ionization (ESI). Analytical HPLC was performed on a Dionex UltiMate HPLC system using a Gemini-NX C18 column (4.6 × 250 mm, 3 μm, 110 Å) with H_2_O:TFA, 100:0.1, v/v (mobile phase A) and MeCN:H_2_O:TFA, 90:10:0.1, v/v/v (mobile phase B) and gradient elution 20–100% B over 15 min or 0–100% B over 15 min. MALDI-HRMS was performed on a Thermo Scientific QExactive Orbitrap mass spectrometer equipped with a TransMIT SMALDI5 ion source. The sample was analyzed in the positive ion mode using a peak from the DHB matrix for internal mass calibration, whereby a mass accuracy of 2 ppm or better was achieved. Optical rotations were recorded on an Anton Paar MCP Polarimeter (Anton Paar Cell 100 mm, CL. 0.01, Ø 5 mm). All test compounds were of ≥95% purity.

### (*S*)-*N-*(1-(1*H*-Tetrazol-5-yl)-3-(4-(trifluoromethyl)phenyl)propan-2-yl)-2-(*m*-tolyl)acetamide (**8**)

Step 1: (*S*)-*N*-(2-Cyanoethyl)-3-(2-(*m*-tolyl)acetamido)-4-(4-(trifluoromethyl)phenyl)butanamide (**6**): A dry vial with **5** (85 mg, 0.22 mmol), HOBt (96 mg, 0.63 mmol), and EDC^.^HCl (108 mg, 0.56 mmol) in DMF (1.1 mL) and DIPEA (156 μL, 0.90 mmol) was added dropwise, and the reaction was stirred under argon at rt for 5 min. Then, 3-aminopropanenitrile (41 μL, 0.56 mmol) was added dropwise and the reaction was stirred at rt until consumption of the starting material. The reaction mixture was extracted with EtOAc, and the organic phase was washed with 1 M HCl_aq_, water, and brine; dried over Na_2_SO_4_; and concentrated in vacuo. The residue was purified by flash chromatography (EtOAc:PE, 1:1 → EtOAc:PE, 1:1 [5% MeOH]) to give 60 mg (62%) of **6** as a white solid: *R_f_* = 0.11 (EtOAc); ^1^H NMR (400 MHz, CD_3_OD): δ 7.47 (d, *J* = 8.0 Hz, 2H), 7.30 (d, *J* = 8.0 Hz, 2H), 7.13 (t, *J* = 7.6 Hz, 1H), 7.04 (d, *J* = 7.6 Hz, 1H), 6.98 (s, 1H), 6.91 (d, *J* = 7.5 Hz, 1H), 4.51–4.42 (m, 1H), 3.40–3.33 (m, 4H), 2.98 (dd, *J* = 13.6, 5.0 Hz, 1H), 2.84 (dd, *J* = 13.8, 9.1 Hz, 1H), 2.58 (t, *J* = 6.6 Hz, 2H), 2.52–2.39 (m, 2H), 2.29 (s, 3H); ^13^C NMR (101 MHz, CD_3_OD: δ 173.6, 173.3, 144.1, 139.3, 136.6, 131.0, 130.8, 129.7 (q, *J* = 32.4 Hz), 129.4, 128.6, 127.1, 126.1 (q, *J* = 3.9 Hz), 125.8 (q, *J* = 271.2 Hz), 119.5, 49.6, 44.0, 41.6, 40.7, 36.5, 21.4, 18.4; HRMS (ESI) calcd for C_23_H_24_F_3_N_3_NaO_2_ (M + Na^+^): 454.1713, found: 454.1725.

Step 2: (*S*)-*N*-(1-(1-(2-Cyanoethyl)-1*H*-tetrazol-5-yl)-3-(4-(trifluoromethyl)phenyl)propan-2-yl)-2-(*m*-tolyl)acetamide (**7**): **6** (77 mg, 0.18 mmol) and Ph_3_P (163 mg, 0.62 mmol) suspended in dry MeCN (1.8 mL) were stirred at 0 °C for 10 min. DIAD (88 μL, 0.45 mmol) was added slowly, and the reaction was stirred for 5 min, after which TMSN_3_ (71 μL, 0.53 mmol) was added and the reaction was stirred at 0 °C for 30 min, then for 2 h at rt, and finally at 50 °C for 2 days. The reaction mixture was cooled to 0 °C, added 3 M aq. NaNO_2_ (175 μL), stirred for 30 min, and then added 0.5 M CAN (500 μL) followed by stirring for additional 30 min. The mixture was diluted with water, and the aqueous phase was extracted with DCM (×3). The combined organic layers were washed with brine, dried over Na_2_SO_4_, concentrated onto Celite, and purified by flash chromatography (1:1, EtOAc:PE → 100% EtOAc→EtOAc [3% MeOH]) to give **7** as a white solid 13 mg (16%) [compound contains 6% triphenylphosphine oxide]): *R_f_* = 0.48 (EtOAc); ^1^H NMR (400 MHz, CD_3_OD): δ 7.50 (d, *J* = 8.0 Hz, 2H), 7.37 (d, *J* = 8.1 Hz, 2H), 7.08 (t, *J* = 7.5 Hz, 1H), 7.02 (d, *J* = 7.6 Hz, 1H), 6.84 (s, 1H), 6.77 (d, *J* = 7.5 Hz, 1H), 4.60–4.55 (m, 2H), 4.54–4.46 (m, 1H), 3.34–3.19 (m, 4H), 3.17 (dd, *J* = 14.0, 4.8 Hz, 1H), 3.13–3.09 (m, 2H), 2.98 (dd, *J* = 13.9, 10.0 Hz, 1H), 2.26 (s, 3H); ^13^C NMR (101 MHz, CD_3_OD): δ 174.0, 154.5, 143.7 (q, *J* = 1.5 Hz), 139.4, 136.3, 130.9, 130.6, 129.9 (q, *J* = 30.5 Hz), 129.5, 128.6, 126.9, 126.3 (q, *J* = 3.8 Hz), 125.8 (q, *J* = 271.0 Hz), 118.1, 50.6, 43.8, 43.8, 40.3, 29.1, 21.4, 18.7; HRMS (ESI) calcd for C_23_H_24_F_3_N_6_O (M + H^+^): 457.1958, found: 457.1977.

Step 3: **7** (13.0 mg, 29 μmol) in THF (200 μL) was added 1.3 M LiOH_aq_ (44 μL, 57 μmol), and the reaction was stirred at rt under argon for 2 h.^55^ The reaction was diluted with water and washed with DCM (×1), and the aqueous phase was acidified (1 M HCl_aq_) until pH 2–3 and extracted with EtOAc (×3). The combined organic layers were washed with brine, dried over Na_2_SO_4_, evaporated onto Celite, and purified by flash chromatography (EtOAc → EtOAc [3% AcOH]) to give **8** as a white solid (7 mg, 61%): *t*_R_ = 11.09 min (purity 100% by HPLC at 254 nm);^1^H NMR (400 MHz, CDCl_3_): δ 7.48 (d, *J* = 8.0 Hz, 2H), 7.32 (d, *J* = 8.0 Hz, 2H), 7.08 (t, *J* = 7.6 Hz, 1H), 7.00 (d, *J* = 7.6 Hz, 1H), 6.86 (s, 1H), 6.78 (d, *J* = 7.5 Hz, 1H), 4.58–4.48 (m, 1H), 3.28 (m, 2H), 3.27–3.15 (m, 2H), 3.05 (dd, *J* = 13.9, 5.1 Hz, 1H), 2.90 (dd, *J* = 13.9, 9.4 Hz, 1H), 2.26 (s, 3H); ^13^C NMR (101 MHz, CDCl_3_): δ 173.8, 155.4, 143.7 (q, *J* = 1.5 Hz), 139.2, 136.4, 130.9, 130.6, 129.8 (q, *J* = 32.3 Hz), 129.4, 128.5, 126.9, 126.2 (q, *J* = 3.9 Hz), 125.8 (q, *J* = 271.1 Hz), 50.7, 43.8, 40.6, 29.6, 21.4; HRMS (ESI) calcd for C_20_H_20_F_3_N_5_NaO (M + Na^+^): 426.1512, found: 426.1503; [α]^20^_D_ = −30° (*c* = 0.02, MeOH).

### (*S*)-*N*-(1-(1*H*-Tetrazol-5-yl)-3-(4-(trifluoromethyl)phenyl)propan-2-yl)-2-phenylacetamide (**16a**)

Step 1: *N*-Boc-4-trifluoromethyl-*L*-phenylalanine (**10**): 4-Trifluoromethyl-l*-*phenylalanine (**9**, 1.50 g, 6.43 mmol) and Boc_2_O (2.27 g, 10.29 mmol) were dissolved in MeOH and H_2_O (1:1, 6.4 mL). The suspension was cooled to 0 °C, and Et_3_N (2.12 mL, 15.25 mmol) was added dropwise. The reaction mixture was heated at 55 °C under argon overnight and then concentrated in vacuo. The residue was dissolved in EtOAc (12 mL) and washed with cold 0.25 mM HCl_aq_. The aqueous phase was further acidified with cold 1 M HCl_aq_ until pH 1 and extracted with EtOAc (×3). The combined organic layers were washed with 0.25 mM HCl_aq_ and brine, dried over MgSO_4_, filtered, and concentrated in vacuo to obtain 2.08 g (97%) of **10** as a pale yellow solid that was used without further purification: *R_f_* = 0.88 (DCM:MeOH, 4:1); ^1^H NMR (400 MHz, CD_3_OD): δ 7.58 (d, *J* = 7.9 Hz, 2H), 7.43 (d, *J* = 7.9 Hz, 2H), 4.40 (dd, *J* = 9.5, 5.0 Hz, 1H), 3.26 (dd, *J* = 13.9, 5.1 Hz, 1H), 2.98 (dd, *J* = 13.8, 9.3 Hz, 1H), 1.36 (s, 9H); ^13^C NMR (151 MHz, CD_3_OD): δ 174.9, 157.8, 143.5, 131.1, 130.0 (q, *J* = 32.2 Hz), 126.2 (q, *J* = 3.9 Hz), 125.8 (q, *J* = 270.9 Hz), 80.6, 55.9, 38.6, 28.6; ESI-MS *m*/*z* 332.1 (M – H^–^); [α]^20^_D_ = +4.8° (*c* = 0.1, MeOH).

Step 2: *tert*-Butyl (*S*)-(1-hydroxy-3-(4-(trifluoromethyl)phenyl)propan-2-yl)carbamate (**11**): A solution of **10** (1.06 g, 3.17 mmol) in dry THF (4 mL) at −15 °C was added *N-*methylmorpholine (NMM, 420 μL, 3.82 mmol) followed by isobutyl chloroformate (500 μL, 3.83 mmol). The reaction mixture was stirred at −15 °C and after 1 h, additional isobutyl chloroformate (250 μL, 1.92 mmol) was added. After 30 min, the precipitate of NMM hydrochloride was filtered off and rinsed with cold THF (16 mL). To the combined filtrates, a solution of NaBH_4_ (180 mg, 4.75 mmol) in water (288 μL) was added (evolution of hydrogen was observed). The reaction mixture was stirred at −15 °C for an additional 1 h and during this time, the evolution of hydrogen ceased. The reaction was quenched with H_2_O (40 mL) and diluted with EtOAc (25 mL) and brine. The aqueous phase was extracted with EtOAc (×3), while the combined organic layers were washed with brine, dried over MgSO_4_, concentrated in vacuo, and purified by flash chromatography (SiO_2_, MeOH in DCM, 5%) to give 652 mg (64%) of **11** as a white solid: *R_f_* = 0.34 (DCM:MeOH, 16:1); ^1^H NMR (400 MHz, CDCl_3_): δ 7.56 (d, *J* = 8.0 Hz, 2H), 7.34 (d, *J* = 7.9 Hz, 2H), 4.76 (d, *J* = 8.3 Hz, 1H), 3.88 (br s, 1H), 3.68 (dd, *J* = 10.9, 3.8 Hz, 1H), 3.56 (dd, *J* = 10.9, 5.0 Hz, 1H), 2.92 (d, *J* = 7.2 Hz, 2H), 1.40 (s, 9H); ^13^C NMR (151 MHz, CDCl_3_): δ 156.0, 142.3, 129.8, 129.1 (q, *J* = 32.4 Hz), 125.6 (q, *J* = 3.7 Hz), 124.4 (q, *J* = 271.9 Hz), 80.0, 64.2, 53.6, 37.4, 28.4; ESI-MS *m*/*z* 220.2 (M-Boc + H ^+^); [α]^20^_D_ = −20.0° (c = 0.1, MeOH).

Step 3: (*S*)-*2*-((*tert*-Butoxycarbonyl)amino)-3-(4-(trifluoromethyl)phenyl)propyl 4-methylbenzenesulfonate (**12**): **11** (642 mg, 2.01 mmol) in dry DCM (5 mL) was added tosyl chloride (498 mg, 2.61 mmol) and DMAP (25 mg, 0.20 mmol), cooled to 0 °C, and added dropwise with dry Et_3_N (700 μL, 5.02 mmol). The reaction mixture was stirred at rt for 3.5 h under an argon atmosphere. The reaction mixture was quenched with saturated aq. NaHCO_3_ (20 mL) and extracted with DCM (×3). The combined organic layers were washed with brine, dried over MgSO_4_, concentrated in vacuo, and purified by flash chromatography (SiO_2_, EtOAc:Hept, 1:9 → 1:6) to give 813 mg (85%) of **12** as a white solid: *R*_*f*_ = 0.71 (EtOAc:Hept, 2:3); ^1^H NMR (400 MHz, CDCl_3_): δ 7.77 (d, *J* = 8.1 Hz, 2H), 7.47 (d, *J* = 7.9 Hz, 2H), 7.35 (d, *J* = 8.0 Hz, 2H), 7.21 (d, *J* = 7.9 Hz, 2H), 4.77 (d, *J* = 8.6 Hz, 1H), 4.13–4.00 (m, 2H), 3.90–3.81 (m, 1H), 3.04–2.75 (m, 2H), 2.46 (s, 3H), 1.37 (s, 9H); ^13^C NMR (151 MHz, CDCl_3_): δ 155.1, 145.4, 141.1, 132.5, 130.2, 129.7, 129.2 (q, *J* = 32.9 Hz), 128.2, 125.6 (q, *J* = 3.8 Hz), 124.3 (q, *J* = 271.9 Hz), 80.2, 70.2, 50.7, 37.2, 28.4, 21.8; ESI-MS *m*/*z* 374.3 (M-Boc + H^+^); [α]^20^_D_ = −17.5° (*c* = 0.1, MeOH). Analysis by chiral HPLC confirmed >99% ee (see the Supporting Information).

Step 4: *tert*-Butyl (*S*)-(1-cyano-3-(4-(trifluoromethyl)phenyl)propan-2-yl)carbamate (**13**): To a round-bottomed flask charged with **12** (1.23 g, 2.60 mmol) in DMF (8 mL) at 0 °C was added NaCN (1.18 g, 24 mmol), and the reaction mixture was stirred at 60 °C for 5 h, then cooled to rt, and diluted with water, EtOAc, and brine. The aqueous phase was extracted with EtOAc (×3), and the combined organic layers were washed with brine, dried over MgSO_4_, concentrated in vacuo, and purified by flash chromatography (SiO_2_, EtOAc:Hept, 1:4) to give 704 mg (82%) of **13** as a white solid: *R*_*f*_ = 0.42 (EtOAc:Hept, 3:7); ^1^H NMR (400 MHz, CDCl_3_): δ 7.60 (d, *J* = 8.0 Hz, 2H), 7.35 (d, *J* = 7.9 Hz, 2H), 4.73 (d, *J* = 8.3 Hz, 1H), 4.12 (br s, 1H), 3.11–3.01 (m, 1H), 3.00–2.90 (m, 1H), 2.78–2.68 (m, 1H), 2.50–2.40 (m, 1H), 1.41 (s, 9H); ^13^C NMR (151 MHz, CDCl_3_): δ 154.9, 140.5, 129.8 (q, *J* = 32.5 Hz), 129.6, 126.0 (q, *J* = 3.6 Hz), 124.2 (q, *J* = 271.9 Hz), 117.2, 80.7, 48.4, 39.5, 28.4, 22.9; ESI-MS *m*/*z* 329.1 (M + H^+^); [α]^20^_D_ = −25.1° (*c* = 0.2, MeOH).

Step 5: (*S*)*-*1-Cyano-3-(4-(trifluoromethyl)phenyl)propan-2-amine hydrochloride (**14**): To a solution of **11** (695 mg, 2.12 mmol) in dry DCM (18 mL) at rt was added dropwise 4 M HCl in dioxane (4.6 mL), and the reaction mixture was stirred overnight and then concentrated in vacuo. The residue was washed with DCM (×3) to give 568 mg of **14** (quantitative) as an off-white solid that was used without further purification: ^1^H NMR (400 MHz, CD_3_OD): δ 7.72 (d, *J* = 8.0 Hz, 2H), 7.53 (d, *J* = 8.0 Hz, 2H), 3.97–3.86 (m, 1H), 3.22–3.12 (m, 2H), 2.97–2.78 (m, 2H); ^13^C NMR (151 MHz, CD_3_OD): δ 140.3, 131.3 (q, *J* = 32.5 Hz), 131.2, 127.3 (q, *J* = 3.9 Hz), 125.6 (q, *J* = 271.3 Hz), 116.4, 49.6, 38.9, 21.6; ESI-MS *m*/*z* 229.1 (M + H^+^); [α]^20^_D_ = +8.6° (*c* = 0.3, MeOH).

Step 6: (*S*)*-N*-(1-Cyano-3-(4-(trifluoromethyl)phenyl)propan-2-yl)-2-phenylacetamide (**15a**): To **14** (27 mg, 0.10 mmol), 2-phenylacetic acid (29 mg, 0.21 mmol), and PyBOP (90 mg, 0.17 mmol) in DMF (0.41 mL) at 0 °C was added dropwise DIPEA (72 μL, 0.41 mmol), and the reaction was stirred at rt overnight. The reaction mixture was diluted with EtOAc (4 mL), and the organic phase was washed with aq. NaHCO_3_ (×3) and brine, dried over MgSO_4_, and concentrated in vacuo to give 25 mg (72%) of **15a** as a white solid after flash chromatography (SiO_2_, EtOAc:Hept, 1:2 → 1:1): *R_f_* = 0.42 (EtOAc:Hept, 1:1): ^1^H NMR (400 MHz, CD_3_OD): δ 7.51 (d, *J* = 8.0 Hz, 2H), 7.34 (d, *J* = 7.9 Hz, 2H), 7.27–7.16 (m, 3H), 7.14–7.07 (m, 2H), 4.45–4.33 (m, 1H), 3.44 (s, 2H), 3.09–3.00 (m, 1H), 2.97–2.87 (m, 1H), 2.86–2.76 (m, 1H), 2.73–2.63 (m, 1H); ^13^C NMR (101 MHz, CD_3_OD): δ 173.9, 143.1, 136.6, 130.9, 130.0, 129.9 (q, *J* = 32.0 Hz), 129.5, 127.9, 126.4 (q, *J* = 3.9 Hz), 125.7 (q, *J* = 270.9 Hz), 118.6, 49.6, 43.7, 39.9, 23.5; ESI-MS *m*/*z* 347.1 (M + H^+^).

Step 7: **15a** (24 mg, 0.07 mmol), NaN_3_ (18 mg, 0.27 mmol), and Et_3_N^.^HCl (38 mg, 0.27 mmol) in toluene (230 μL) under argon were stirred overnight at reflux. The reaction mixture was cooled to 0 °C and added 1 M HCl_aq_ until pH 1 (NB: Take care at a larger scale. Toxic and explosive hydrazoic acid may form. Aqueous solutions were basified and/or quenched with oxidant before disposal). The aqueous phase was extracted with EtOAc (×4). The combined organic layers were washed with brine, dried over MgSO_4_, and concentrated in vacuo to give 15 mg (58%) of **16a** as a white solid after purification by flash chromatography (SiO_2_, EtOAc:Hept, 3:2 then MeOH/DCM/AcOH, 5:95:1): *t_R_* = 10.27 min (purity 99.07% by HPLC at 220 nm); ^1^H NMR (400 MHz, CD_3_OD): δ 7.49 (d, *J* = 8.0 Hz, 2H), 7.34 (d, *J* = 7.9 Hz, 2H), 7.24–7.13 (m, 3H), 7.02–6.95 (m, 2H), 4.61–4.47 (m, 1H), 3.37–3.32 (m, 2H), 3.26–3.15 (m, 2H), 3.09–3.01 (m, 1H), 2.95–2.83 (m, 1H); ^13^C NMR (151 MHz, CD_3_OD): δ 173.7, 155.5, 143.7, 136.6, 130.9, 130.1 (q, *J* = 32.0 Hz), 129.9, 129.5, 127.8, 126.3 (q, *J* = 3.8 Hz), 124.0 (q, *J* = 270.7 Hz), 50.8, 43.8, 40.6, 29.6; ESI-MS *m*/*z* 390.2 (M + H^+^); HRMS calcd for C_19_H_19_F_3_N_5_O^+^ (M + H^+^): 390.1536, found: 390.1534; [α]^20^_D_ = −2.1° (*c* = 0.1, MeOH).

### *N*-((*S*)-1-(1*H*-Tetrazol-5-yl)-3-(4-(trifluoromethyl)phenyl)propan-2-yl)bicyclo[4.2.0]octa-1(6),2,4-triene-7-carboxamide (16b)

Step 1: **14** (30 mg, 0.11 mmol) was coupled with bicyclo[4.2.0]octa-1(6),2,4-triene-7-carboxylic acid (35 mg, 0.24 mmol) using PyBOP (100 mg, 0.19 mmol) and DIPEA (80 μL, 0.46 mmol) as described for **15a** to give 31 mg (77%) of **15b** as a white solid after flash chromatography (SiO_2_, EtOAc:Hept, 0:1 → 1:1): *R_f_* = 0.46 (EtOAc:Hept, 1:1); ^1^H NMR assigned as mixture of two diastereomers (400 MHz, CDCl_3_): δ 7.63–7.47 (m, 2H), 7.34–7.27 (m, 2H), 7.24–7.06 (m, 3H), 7.06–6.89 (m, 1H), 5.83–5.54 (m, 1H), 4.44–4.33 (m, 1H), 4.21–4.08 (m, 1H), 3.67–3.36 (m, 2H), 3.30–2.91 (m, 2H), 2.86–2.74 (m, 1H), 2.64–2.47 (m, 1H); ^13^C NMR assigned as mixture of two diastereomers (151 MHz, CDCl_3_): δ 172.5, 172.2, 144.8, 144.8, 141.9, 141.9, 140.1, 140.1, 129.5, 129.4, 129.0, 128.1, 128.0, 127.3, 126.1, 126.1, 126.0, 126.0, 123.9, 123.8, 122.3, 122.2, 117.0, 116.9, 47.8, 47.7, 47.1, 46.9, 45.7, 38.9, 38.8, 35.7, 35.6, 22.9, 22.5; ESI-MS *m*/*z* 359.2 (M + H^+^).

Step 2: **15b** (20 mg, 0.06 mmol) was reacted with NaN_3_ (12 mg, 0.18 mmol) as described for **16a** to give 20 mg (90%) of **16b** as a pale yellow solid after trituration with heptane: *t_R_* = 10.63 min (purity 95.46% by HPLC at 254 nm); ^1^H NMR assigned as a mixture of two diastereomers (400 MHz, CD_3_OD): δ 7.60–7.52 (m, 2H), 7.46–7.38 (m, 2H), 7.22–7.06 (m, 2H), 7.05–6.98 (m, 1H), 6.94–6.62 (m, 1H), 4.67–4.52 (m, 1H), 4.07–4.00 (m, 1H), 3.30–3.19 (m, 3H), 3.15–2.87 (m, 3H); ^13^C NMR assigned as mixture of two diastereomers (151 MHz, CD_3_OD): δ 174.3, 174.2, 155.6, 145.7, 145.6, 144.53, 144.47, 143.9, 131.04, 130.98, 129.9 (m), 129.10, 129.08, 128.3, 128.1, 126.3 (m), 125.8 (m), 123.73, 123.70, 123.3, 123.0, 50.8, 50.6, 48.1, 48.0, 40.8, 40.7, 34.6, 34.3, 29.7, 29.4; ESI-MS *m*/*z* 402.1 (M + H^+^); HRMS calcd for C_20_H_19_F_3_N_5_O^+^ (M + H^+^): 402.1536, found: 402.1537; [α]^20^_D_ = −6.8° (*c* = 0.4, MeOH).

### (*S*)*-N-(*1-(1*H*-Tetrazol-5-yl)-3-(4-(trifluoromethyl)phenyl)propan-2-yl)-2-(3-fluorophenyl)acetamide (**16c**)

Step 1: **14** (23 mg, 0.09 mmol) was coupled with 2-(3-fluorophenyl)acetic acid (28 mg, 0.18 mmol) as described for **15a** to give 14 mg (43%) of **15c** as a white solid after flash chromatography (SiO_2_, EtOAc:Hept, 1:2 → 1:1): *R_f_*= 0.35 (EtOAc:Hept, 1:1); ^1^H NMR (400 MHz, CD_3_OD): δ 7.51 (d, *J* = 8.0 Hz, 2H), 7.35 (d, *J* = 7.9 Hz, 2H), 7.28–7.18 (m, 1H), 6.98–6.85 (m, 3H), 4.45–4.34 (m, 1H), 3.44 (s, 2H), 3.09–3.00 (m, 1H), 2.97–2.87 (m, 1H), 2.87–2.77 (m, 1H), 2.74–2.63 (m, 1H); ^13^C NMR (151 MHz, CD_3_OD): δ 173.2, 164.2 (d, *J* = 244.5 Hz), 143.1, 139.2 (d, *J* = 8.0 Hz), 131.1 (d, *J* = 8.2 Hz), 130.9, 130.1 (q, *J* = 32.2 Hz), 126.3 (q, *J* = 3.7 Hz), 125.8 (d, *J* = 2.8 Hz), 125.7 (q, *J* = 271.1 Hz), 118.6, 116.8 (d, *J* = 21.9 Hz), 114.6 (d, *J* = 21.3 Hz), 49.6, 43.3, 39.9, 23.5; ESI-MS *m*/*z* 365.2 (M + H^+^); [α]^20^_D_ = +10.2° (*c* = 0.1, MeOH).

Step 2: **15c** (13 mg, 0.04 mmol) was reacted with NaN_3_ (14 mg, 0.22 mmol) and Et_3_N^.^HCl (30 mg, 0.22 mmol) in toluene (290 μL) as described for **16a** to give 7 mg (45%) of **16c** as a white solid after purification by flash chromatography (SiO_2_, EtOAc:Hept, 3:2 then MeOH/DCM/AcOH, 5:95:1): *t_R_* = 10.43 min (purity 96.93% by HPLC at 220 nm); ^1^H NMR (400 MHz, CD_3_OD): δ 7.48 (d, *J* = 7.9 Hz, 2H), 7.33 (d, *J* = 7.9 Hz, 2H), 7.23–7.14 (m, 1H), 6.96–6.86 (m, 1H), 6.83–6.75 (m, 2H), 4.54 (s, 1H), 3.42–3.33 (m, 2H), 3.24–3.13 (m, 2H), 3.05–2.98 (m, 1H), 2.89–2.78 (m, 1H); ^13^C NMR (151 MHz, CD_3_OD): δ 172.9, 164.1 (d, *J* = 244.6 Hz), 156.6, 143.9, 139.3 (d, *J* = 7.8 Hz), 131.0 (d, *J* = 8.4 Hz), 130.9, 129.8 (q, *J* = 32.2 Hz), 126.2 (q, *J* = 3.7 Hz), 125.8 (q, *J* = 270.7 Hz), 125.7 (d, *J* = 2.8 Hz), 116.8 (d, *J* = 22.0 Hz), 114.5 (d, *J* = 21.2 Hz), 51.0, 43.4, 40.6, 30.1; ESI-MS *m*/*z* 408.3 (M + H^+^); HRMS calcd for C_19_H_18_F_4_N_5_O^+^ (M + H^+^): 408.1442, found: 408.1441; [α]^20^_D_ = +10.1° (*c* = 0.1, MeOH).

### (*S*)*-*2-(3-Fluorophenyl)-*N*-(1-phenyl-3-(1*H*-tetrazol-5-yl)propan-2-yl)acetamide (**16d**)

Step 1: (*S*)-2-((*tert*-Butoxycarbonyl)amino)-3-phenylpropyl 4-methylbenzenesulfonate^46^ was reacted with NaCN (1.1 g, 22 mmol) as described for **13**. Purification by flash chromatography (8% →15% EtOAc in Hept) afforded 464 mg (72%) of *tert*-butyl (*S*)-(1-cyano-3-phenylpropan-2-yl)carbamate as a white powder: *R_f_* = 0.27 (EtOAc:Hept, 2:8); ^1^H NMR (400 MHz, CDCl_3_): δ 7.39–7.20 (m, 5H), 4.77 (d, *J* = 8.1 Hz, 1H), 4.11 (s, 1H), 3.03 (dd, *J* = 13.7, 6.5 Hz, 1H), 2.89 (dd, *J* = 13.8, 8.3 Hz, 1H), 2.77–2.67 (m, 1H), 2.45 (dd, *J* = 16.8, 4.4 Hz, 1H), 1.46 (s, 9H); ^13^C NMR (101 MHz, CDCl_3_): δ 155.0, 136.3, 129.3, 129.1, 127.4, 117.4, 80.5, 48.7, 39.6, 28.4, 22.6. Spectra are in agreement with the literature reported data.^56^

Step 2: *tert*-Butyl (*S*)-(1-cyano-3-phenylpropan-2-yl)carbamate (150 mg, 0.57 mmol) was deprotected as described for **14**. The crude product was coupled with 2-(3-fluorophenyl)acetic acid (117 mg, 0.75 mmol) as described for **15a**. The residue was purified by flash chromatography (20% → 28% EtOAc in Hept) to give 101 mg (59% over two steps) of (*S*)*-N*-(1-cyano-3-phenylpropan-2-yl)-2-(3-fluorophenyl)acetamide (**15d**) as a white solid: *R_f_* = 0.33 (EtOAc:Hept, 1:1); ^1^H NMR (400 MHz, CDCl_3_): δ 7.32–7.22 (m, 4H), 7.08–7.03 (m, 2H), 7.02–6.96 (m, 1H), 6.95–6.90 (m, 1H), 6.87–6.81 (m, 1H), 5.53 (d, *J* = 7.5 Hz, 1H), 4.37–4.23 (m, 1H), 3.50 (s, 2H), 2.87 (d, *J* = 7.5 Hz, 2H), 2.74 (dd, *J* = 16.8, 5.6 Hz, 1H), 2.48 (dd, *J* = 16.8, 4.2 Hz, 1H); ^13^C NMR (101 MHz, CDCl_3_): δ 170.4, 163.2 (d, *J* = 247.5 Hz), 136.5 (d, *J* = 7.4 Hz), 135.7, 130.8 (d, *J* = 8.4 Hz), 129.1, 129.0, 127.6, 125.2 (d, *J* = 3.0 Hz), 117.2, 116.5 (d, *J* = 21.6 Hz), 114.8 (d, *J* = 21.0 Hz), 47.5, 43.4 (d, *J* = 1.8 Hz), 38.9, 22.4; m.p. 124.0–126.4 °C ; MS (*m*/*z*) for C_18_H_17_FN_2_O calcd: 296.13 found (M + H^+^): 297.2; [α]^20^_D_ = −13.1° (*c =* 0.2, MeOH).

Step 3: (*S*)*-N*-(1-Cyano-3-phenylpropan-2-yl)-2-(3-fluorophenyl)acetamide (100 mg, 0.32 mmol) was reacted with NaN_3_ (84 mg, 1.29 mmol) as described for **16a** and purified by flash column chromatography (0% → 2% MeOH in DCM + 1% AcOH) to give 75 mg (68%) of **16d** as a white solid: *R_f_* = 0.29 (MeOH/AcOH/DCM, 5:1:94); ^1^H NMR (600 MHz, CD_3_OD): δ 7.25–7.16 (m, 6H), 6.93–6.89 (m, 1H), 6.81–6.76 (m, 2H), 4.51 (tt, *J* = 8.6, 5.5 Hz, 1H), 3.38–3.33 (m, 2H), 3.21 (dd, *J* = 14.9, 5.3 Hz, 1H), 3.14 (dd, *J* = 14.9, 8.3 Hz, 1H), 2.95 (dd, *J* = 13.8, 5.7 Hz, 1H), 2.82 (dd, *J* = 13.8, 8.8 Hz, 1H); ^13^C NMR (151 MHz, CD_3_OD): δ 172.9, 164.1 (d, *J* = 244.3 Hz), 155.5, 139.2 (d, *J* = 7.7 Hz), 138.9, 131.1 (d, *J* = 8.5 Hz), 130.3, 129.5, 127.7, 125.8 (d, *J* = 2.9 Hz), 116.8 (d, *J* = 21.9 Hz), 114.5 (d, *J* = 21.4 Hz), 51.1, 43.3 (d, *J* = 1.7 Hz), 50.0, 29.5; m.p. 184.2–191.2 °C; HRMS calcd for C_18_H_19_FN_5_O^+^ (M + H^+^): 340.1568, found: 340.1567; [α]^20^_D_ = −9.3° (*c =* 0.2, MeOH).

### (*S*)-*N*-(1-(1*H*-Tetrazol-5-yl)-3-(4-(trifluoromethyl)phenyl)propan-2-yl)-2-(3-chlorophenyl)acetamide (**16e**)

Step 1: **14** (24 mg, 0.11 mmol) was coupled with 2-(3-chlorophenyl)acetic acid (38 mg, 0.22 mmol) as described for **15a** to give 28 mg (68%) of **15e** as a white solid after flash chromatography (SiO_2_, EtOAc:PE, 1:9 → 1:4): *R_f_* = 0.53 (EtOAc:PE, 1:1); ^1^H NMR (600 MHz, CD_3_OD): δ 7.52 (d, *J* = 8.0 Hz, 2H), 7.35 (d, *J* = 7.8 Hz, 2H), 7.25–7.17 (m, 3H), 7.07–7.01 (m, 1H), 4.42–4.35 (m, 1H), 3.44 (s, 2H), 3.07–3.00 (m, 1H), 2.96–2.88 (m, 1H), 2.85–2.78 (m, 1H), 2.73–2.65 (m, 1H); ^13^C NMR (151 MHz, CD_3_OD): δ 173.1, 143.1, 138.8, 135.2, 130.9, 130.8, 130.14, 130.10 (q, *J* = 32.2 Hz), 128.4, 128.0, 126.4 (q, *J* = 3.9 Hz), 125.7 (q, *J* = 270.9 Hz), 118.6, 49.6, 43.2, 39.9, 23.5; ESI-MS *m*/*z* 381.1 (M + H^+^); HRMS calcd for C_19_H_17_ClF_3_N_2_O^+^ (M + H^+^): 381.0976, found: 381.0974; [α]^20^_D_ = −7.0° (*c* = 0.1, MeOH).

Step 2: **15e** (28 mg, 0.07 mmol), NaN_3_ (20 mg, 0.29 mmol), and Et_3_N^.^HCl (41 mg, 0.29 mmol) in dry toluene (250 μL) were stirred under argon at reflux. After 2 days, the reaction was not complete, thus additional NaN_3_ (9.5 mg, 0.14 mmol) and Et_3_N^.^HCl (20 mg, 0.14 mmol) were added and the reaction mixture was stirred for additional 12 h at reflux. The reaction was worked up as described for **15a** to give 12 mg (39%) of **16e** as a white solid after purification by flash chromatography (SiO_2_, PE/EtOAc, 1:1 then MeOH/DCM/AcOH, 5:94:1): *R*_*f*_ = 0.26 (MeOH/DCM/AcOH, 5:94:1); *t_R_* = 10.96 min (purity 97.62% by HPLC at 220 nm); ^1^H NMR (600 MHz, CD_3_OD): δ 7.49 (d, *J* = 8.0 Hz, 2H), 7.33 (d, *J* = 8.0 Hz, 2H), 7.22–7.15 (m, 2H), 7.12 (t, *J* = 1.8 Hz, 1H), 6.91 (dt, *J* = 7.3, 1.6 Hz, 1H), 4.59–4.45 (m, 1H), 3.36–3.32 (m, 1H), 3.30–3.28 (m, 1H), 3.27–3.22 (m, 1H), 3.21–3.15 (m, 1H), 3.08–3.02 (m, 1H), 2.91–2.84 (m, 1H); ^13^C NMR (151 MHz, CD_3_OD): δ 172.9, 155.8, 143.7, 138.8, 135.2, 130.9, 130.1, 129.9 (q, *J* = 32.2 Hz), 128.3, 127.9, 126.2 (q, *J* = 3.9 Hz), 125.8 (q, *J* = 271.0 Hz), 50.9, 43.3, 40.6, 29.8; ESI-MS *m*/*z* 424.1 (M + H^+^); HRMS calcd for C_19_H_18_ClF_3_N_5_O^+^ (M + H^+^): 424.1146, found: 424.1146; [α]^20^_D_ = −13.5° (*c* = 0.1, MeOH).

### (*R*)-*N*-(4-(1*H*-Tetrazol-5-yl)-1-(4-(trifluoromethyl)phenyl)butan-2-yl)-2-(3-chlorophenyl)-acetamide (**21**)

Step 1: **17**(43) (382 mg, 1.10 mmol) and 1 M DIBALH in toluene (2.20 mL, 2.20 mmol) were stirred under argon at −78 °C for 80 min, then quenched with saturated Rochelle’s salt, and extracted with EtOAc (×3). The combined organic layers were dried over Na_2_SO_4_, washed with brine, and concentrated in vacuo to yield the aldehyde. The crude aldehyde was reacted in two batches with KO*t*Bu (2 equiv) and freshly prepared (cyanomethyl)triphenylphosphonium bromide (2 equiv) at rt overnight to produce the crude Wittig product. A solution of the combined crude Wittig products in EtOH and Pd/C (10% by weight) was stirred under H_2_ at rt overnight. After complete consumption of starting material, the mixture was filtered through a Celite pad, concentrated in vacuo, and purified by flash chromatography (SiO_2_, EtOAc:PE 1:3) to give 145 mg (39% over two steps) of **18** as a white solid: *R_f_* = 0.23 (EtOAc:PE 1:2); ^1^H NMR (400 MHz, DMSO-*d*_6_): δ 7.57 (d, *J* = 7.8 Hz, 2H), 7.30 (d, *J* = 7.9 Hz, 2H), 4.34 (d, *J* = 9. Hz, 1H), 3.97–3.82 (m, 1H), 2.98–2.81 (m, 2H), 2.52–2.34 (m, 2H), 1.93 (dtd, *J* = 15.5, 7.9, 3.6 Hz, 1H), 1.83–1.65 (m, 1H), 1.38 (s, 9H); ^13^C NMR (101 MHz, DMSO-*d*_6_): δ 155.5, 141.5, 129.8, 129.6–129.2 (m), 125.7 (q, *J* = 3.7 Hz), 123.5–120.0 (m), 119.4, 80.2, 51.4, 41.4, 30.7, 28.4, 14.5; ESI-HRMS calcd for C_17_H_21_F_3_N_2_O_2_Na (M + Na^+^): 365.1447, found: 365.1433.

Step 2: **18** (96 mg, 280 μmol) at 0 °C was added pre-cooled 4 M HCl in dioxane (5.0 mL, 20.0 mmol). The ice bath was removed, and the reaction was stirred at rt until the consumption of the starting material as followed by TLC. The reaction mixture was concentrated in vacuo and the residue dried on high vacuum to afford 78 mg (quant) of **19** as brown oil that was used directly in the next step: *R_f_* = 0.07 (EtOAc [5% MeOH]); ESI-HRMS calcd for C_12_H_14_F_3_N_2_ (M + H^+^): 243.1104, found: 243.1098.

Step 3: **19** (78 mg, 280 μmol) and 2-(3-chlorophenyl)acetic acid (74 mg, 434 μmol) was coupled as described for **15a** and purified by flash chromatography (SiO_2_, EtOAc:PE 1:1) to give 71 mg (64%) of **20** as a white solid: *R_f_* = 0.10 (EtOAc:PE 1:1); ^1^H NMR (400 MHz, CDCl_3_): δ 7.51 (d, *J* = 8.0 Hz, 2H), 7.32–7.21 (m, 2H), 7.16–7.10 (m, 3H), 7.02–6.96 (m, 1H), 5.18 (d, *J* = 8.8 Hz, 1H), 4.33–4.19 (m, 1H), 3.48 (s, 2H), 2.90–2.75 (m, 2H), 2.46–2.29 (m, 2H), 1.94 (dtd, *J* = 14.3, 7.2, 3.9 Hz, 1H), 1.76 (ddt, *J* = 14.2, 10.3, 7.2 Hz, 1H); ^13^C NMR (101 MHz, CDCl_3_): δ 170.5, 140.9, 136.4, 135.0, 130.5, 129.6, 129.5, 129.8–129.3 (m), 128.0, 127.6, 125.8 (q, *J* = 3.79 Hz), 125.6–122.6 (m), 119.5, 50.1, 43.5, 40.5, 30.4, 14.6; ESI-HRMS calcd for C_20_H_18_ClF_3_N_2_ONa (M + Na^+^): 417.0952, found: 417.0950; HPLC: *t_R_* = 12.06 min, purity 99.73%; [α]^20^_D_ = −4.5° (*c* = 0.2, MeOH).

Step 4: **20** (30 mg, 76 μmol), NaN_3_ (43 mg, 658 μmol), ZnBr_2_ (44 mg, 196 μmol), and 0.7 mL H_2_O:*i*PrOH (1:1) were stirred at 130 °C (νμ) for 2.5 h. The reaction mixture was acidified with 1 M HCl_aq_ and extracted with EtOAc (×3). The combined organic layers were washed with brine, dried over Na_2_SO_4_, concentrated in vacuo, and purified by flash chromatography (SiO_2_, DCM[2% MeOH+0.1%AcOH] → DCM[5% MeOH+0.1%AcOH]) to give 6 mg (17%) of **21** as a white solid: ^1^H NMR (400 MHz, CD_3_OD): δ 7.36 (d, *J* = 8.0 Hz, 2H), 7.19 (d, *J* = 8.0 Hz, 2H), 7.14–7.07 (m, 3H), 6.95–6.89 (m, 1H), 4.12–4.03 (m, 1H), 3.28 (s, 2H), 2.95–2.78 (m, 3H), 2.69 (dd, *J* = 13.8, 9.1 Hz, 1H), 2.07–1.95 (m, 1H), 1.91–1.79 (m, 1H); ^13^C NMR (101 MHz, CD_3_OD): δ 173.1, 158.2, 144.1, 139.0, 135.2, 130.9, 130.9, 130.2, 130.3–129.2 (m), 129.8, 129.5, 129.2, 128.3, 128.0, 126.1 (q, *J* = 3.8 Hz), 127.2–124.2 (m), 51.4, 43.5, 41.5, 33.7, 21.5; ESI-HRMS calcd for C_20_H_19_ClF_3_N_5_ONa (M + Na^+^): 460.1122, found: 460.1140; HPLC: *t_R_* = 11.28 min, purity 97.19%; [α]^20^_D_ = −9.0° (c = 0.2, MeOH).

### (*S*)*-N-(*1-(1*H*-Tetrazol-5-yl)-3-(4-(trifluoromethyl)phenyl)propan-2-yl)-2-(4-chlorophenyl)acetamide (**16f**)

Step 1: **14** (20 mg, 0.07 mmol) was coupled with 2-(4-chlorophenyl)acetic acid (27 mg, 0.16 mmol) as described for **15a** to give 20 mg (71%) of **15f** as a white solid after flash chromatography (SiO_2_, EtOAc:Hept, 1:2): *R_f_* = 0.38 (EtOAc:Hept, 1:1); ^1^H NMR (400 MHz, CD_3_OD): δ 7.52 (d, *J* = 8.0 Hz, 2H), 7.35 (d, *J* = 7.9 Hz, 2H), 7.22 (d, *J* = 8.4 Hz, 2H), 7.08 (d, *J* = 8.5 Hz, 2H), 4.48–4.34 (m, 1H), 3.41 (s, 2H), 3.09–3.00 (m, 1H), 2.96–2.86 (m, 1H), 2.86–2.76 (m, 1H), 2.74–2.63 (m, 1H); ^13^C NMR (101 MHz, CD_3_OD): δ 173.3, 143.1, 135.4, 133.8, 131.5, 130.9, 130.1 (q, *J* = 32.2 Hz), 129.5, 126.3 (q, *J* = 3.7 Hz), 125.7 (q, *J* = 271.0 Hz), 118.6, 49.6, 42.9, 40.0, 23.5; ESI-MS *m*/*z* 381.1 (M + H^+^); [α]^20^_D_ = −23.3° (*c* = 0.1, MeOH).

Step 2: **15f** (20 mg, 0.05 mmol) was reacted with NaN_3_ (14 mg, 0.21 mmol) as described for **16a** to give 5 mg (23%) of **16f** as a white solid after purification by flash chromatography (SiO_2_, EtOAc:Hept, 3:2 then MeOH/DCM/AcOH, 5:94:0.1): *t_R_* = 11.00 min (purity 98.70% by HPLC at 220 nm); ^1^H NMR (400 MHz, CD_3_OD): δ 7.50 (d, *J* = 8.0 Hz, 2H), 7.34 (d, *J* = 8.0 Hz, 2H), 7.18 (d, *J* = 8.4 Hz, 2H), 6.95 (d, *J* = 8.4 Hz, 2H), 4.54 (dq, *J* = 13.6, 5.4 Hz, 1H), 3.25–3.13 (m, 2H), 3.05 (dd, *J* = 13.9, 5.1 Hz, 1H), 2.87 (dd, *J* = 13.9, 9.5 Hz, 1H); ^13^C NMR (151 MHz, CD_3_OD): δ 173.1, 155.9, 143.8, 135.4, 133.7, 131.4, 130.9, 129.9 (q, *J* = 32.0 Hz), 129.5, 126.2 (q, *J* = 3.8 Hz), 125.8 (q, *J* = 271.2 Hz), 50.9, 43.0, 40.7, 29.8; ESI-MS *m*/*z* 424.3 (M + H^+^); HRMS calcd for C_19_H_18_ClF_3_N_5_O^+^ (M + H^+^): 424.1146, found: 424.1146; [α]^20^_D_ = −2.8° (*c* = 0.2, MeOH).

### (*S*)-*N-*(1-(1*H*-Tetrazol-5-yl)-3-(4-(trifluoromethyl)phenyl)propan-2-yl)-2-(3-bromophenyl)acetamide (**16g**)

Step 1: **14** (30 mg, 0.11 mmol) was coupled with 2-(3-chlorophenyl)acetic acid (51 mg, 0.24 mmol) as described for **15a** to give 40 mg (84%) of **15 g** as a white solid after flash chromatography (SiO_2_, EtOAc:Hept, 1:2 → 1:1): *R_f_* = 0.33 (EtOAc:PE, 1:1); ^1^H NMR (400 MHz, CDCl_3_): δ 7.55 (d, *J* = 8.0 Hz, 2H), 7.44 (dt, *J* = 7.9, 1.5 Hz, 1H), 7.35–7.31 (m, 1H), 7.22–7.12 (m, 3H), 7.06 (dt, *J* = 7.6, 1.4 Hz, 1H), 5.60 (d, *J* = 7.7 Hz, 1H), 4.59–4.23 (m, 1H), 3.48 (s, 2H), 2.96 (d, *J* = 7.7 Hz, 1H), 2.77 (dd, *J* = 16.9, 5.5 Hz, 1H), 2.52 (dd, *J* = 16.9, 4.4 Hz, 1H); ^13^C NMR (151 MHz, CDCl_3_): δ 170.3, 139.9, 136.3, 132.5, 131.0, 130.8, 130.0 (q, *J* = 32.5 Hz), 129.4, 127.9, 126.1 (q, *J* = 3.6 Hz), 124.09 (q, *J* = 270.9 Hz), 123.2, 116.9, 47.3, 43.2, 38.7, 22.7; ESI-MS *m*/*z* 425.1 (M + H^+^); [α]^20^_D_ = −32.8° (c = 0.3, MeOH).

Step 2: **15g** was reacted with NaN_3_ (14 mg, 0.22 mmol) as described for **16a** to give 4 mg (16%) of **16g** as a white solid after purification by flash chromatography (SiO_2_, EtOAc:MeOH, 100:0 → 80:20): *t_R_* = 11.14 min (purity 99.46% by HPLC at 220 nm); ^1^H NMR (600 MHz, CD_3_OD): δ 7.48 (d, *J* = 8.0 Hz, 2H), 7.35 (d, *J* = 7.9 Hz, 1H), 7.32–7.29 (m, 3H), 7.11 (t, *J* = 7.8 Hz, 1H), 6.97 (d, *J* = 8.3 Hz, 1H), 4.57–4.47 (m, 1H), 3.36–3.31 (m, 2H), 3.24–3.19 (m, 1H), 3.18–3.13 (m, 1H), 3.05–2.99 (m, 1H), 2.88–2.80 (m, 1H); ^13^C NMR (151 MHz, CD_3_OD): δ 172.7, 157.0, 143.9, 139.2, 133.1, 131.2, 130.94, 130.91, 129.8 (q, *J* = 32.2 Hz), 128.7, 126.2 (q, *J* = 3.9 Hz), 125.8 (q, *J* = 270.9 Hz), 123.2, 51.1, 43.3, 40.5, 30.1; ESI-MS *m*/*z* 469.0 (M + H^+^); HRMS calcd for C_19_H_18_BrF_3_N_5_O^+^ (M + H^+^): 468.0641, found: 468.0644; [α]^20^_D_ = −15.7° (c = 0.1, MeOH).

### (*S*)-*N*-(1-(1*H*-Tetrazol-5-yl)-3-(4-(trifluoromethyl)phenyl)propan-2-yl)-2-(3-iodophenyl)acetamide (**16h**)

Step 1: **14** (30 mg, 0.11 mmol) was coupled with 2-(3-iodophenyl)acetic acid (62 mg, 0.24 mmol) as described for **15a** to give 46 mg (86%) of **15 h** as a white solid after flash chromatography (SiO_2_, EtOAc:Hept, 0:100 → 1:1): *R_f_* = 0.32 (EtOAc:Hept, 1:1); ^1^H NMR (400 MHz, CDCl_3_): δ 7.67–7.62 (m, 1H), 7.58–7.52 (m, 3H), 7.21 (d, *J* = 8.0 Hz, 2H), 7.12–6.98 (m, 2H), 5.65 (d, *J* = 7.8 Hz, 1H), 4.43–4.31 (m, 1H), 3.45 (s, 2H), 3.05–2.90 (m, 2H), 2.81–2.74 (m, 1H), 2.59–2.43 (m, 1H); ^13^C NMR (151 MHz, CDCl_3_): δ 170.4, 139.9, 138.3, 136.9, 136.4, 130.9, 129.9 (q, *J* = 32.5 Hz), 129.4, 128.5, 126.1 (q, *J* = 3.9 Hz), 124.1 (q, *J* = 272.0 Hz), 117.0, 95.0, 47.3, 43.0, 38.7, 22.7; ESI-MS *m*/*z* 473.1 (M + H^+^); [α]^20^_D_ = −31.6° (*c* = 0.5, MeOH).

Step 2: **15h** (20 mg, 0.04 mmol) was reacted with NaN_3_ (11 mg, 0.17 mmol) as described for **16a** to give 12 mg (54%) of **16h** as a white solid after purification by flash chromatography (SiO_2_, EtOAc:MeOH, 100:0 → 80:20): *t_R_* = 11.36 min (purity 97.68% by HPLC at 220 nm); ^1^H NMR (600 MHz, DMSO-*d*_6_): δ 8.15 (d, *J* = 8.5 Hz, 1H, N*H*), 7.62–7.53 (m, 3H), 7.51 (br s, 1H), 7.36 (d, *J* = 7.9 Hz, 2H), 7.03–6.91 (m, 2H), 4.43–4.25 (m, 1H), 3.29–3.19 (m, 2H), 3.17–3.12 (m, 1H), 3.10–3.04 (m, 1H), 2.98–2.90 (m, 1H), 2.83–2.75 (m, 1H); ^13^C NMR (151 MHz, DMSO-*d*_6_): δ 169.1, 143.1, 138.7, 137.6, 135.0, 130.1, 129.9, 128.2, 126.9 (q, *J* = 31.8 Hz), 124.8 (q, *J* = 3.9 Hz), 124.4 (q, *J* = 271.9 Hz), 94.4, 48.7, 41.6, 38.8, 28.3; ESI-MS *m*/*z* 516.0 (M + H^+^); HRMS calcd for C_19_H_18_F_3_IN_5_O^+^ (M + H^+^): 516.0502, found: 516.0506; [α]^20^_D_ = −11.3° (*c* = 0.1, MeOH).

### (*S*)-*N*-(1-(1*H*-Tetrazol-5-yl)-3-(4-(trifluoromethyl)phenyl)propan-2-yl)-2-(3-(trifluoromethyl)phenyl)acetamide (**16i**)

Step 1: **14** (30 mg, 0.11 mmol) was coupled with 2-(3-(trifluoromethyl)phenyl)acetic acid (49 mg, 0.24 mmol) as described for **15a** to give **15i** (37 mg, 78%) as a white solid after purification by automated flash chromatography (SiO_2_, EtOAc:Hept, 0:100 → 1:1): *R_f_* = 0.26 (EtOAc:Hept, 1:1); ^1^H NMR (400 MHz, CD_3_OD): δ 7.55–7.47 (m, 4H), 7.42 (t, *J* = 8.0 Hz, 1H), 7.38–7.34 (m, 3H), 4.45–4.35 (m, 1H), 3.53 (s, 2H), 3.04 (dd, *J* = 13.9, 5.3 Hz, 1H), 2.92 (dd, *J* = 14.0, 9.6 Hz, 1H), 2.82 (dd, *J* = 17.0, 5.1 Hz, 1H), 2.68 (dd, *J* = 17.0, 7.0 Hz, 1H); ^13^C NMR (101 MHz, CD_3_OD): δ 172.9, 143.1, 137.9, 131.8 (q, *J* = 32.1 Hz), 130.1 (q, *J* = 32.2 Hz), 133.7, 130.8, 130.2, 125.7 (q, *J* = 270.8 Hz), 125.6 (q, *J* = 271.3 Hz), 26.8 (q, *J* = 3.9 Hz), 126.3 (q, *J* = 3.8 Hz), 124.7 (q, *J* = 3.9 Hz), 118.6, 48.7, 43.2, 39.9, 23.5; ESI-MS *m*/*z* 415.5 (M + H^+^); [α]^20^_D_ = −23.5° (*c* = 0.3, MeOH).

Step 2: **15i** (22 mg, 0.05 mmol) was reacted with NaN_3_ (14 mg, 0.21 mmol), Et_3_N^.^HCl (30 mg, 0.22 mmol) as described for **16a** to give 12 mg (48%) of **16i** as a white solid after purification by flash chromatography (SiO_2_, DCM:MeOH, 95:5 → 9:1 + 1% AcOH): *t_R_* = 11.16 min (purity 99.46% by HPLC at 220 nm); ^1^H NMR (600 MHz, CD_3_OD): δ 7.49 (d, *J* = 7.8 Hz, 1H), 7.47–7.43 (m, 3H), 7.38 (t, *J* = 7.7 Hz, 1H), 7.32 (d, *J* = 8.0 Hz, 2H), 7.24 (d, *J* = 7.7 Hz, 1H), 4.58–4.50 (m, 1H), 3.48–3.35 (m, 2H), 3.26–3.12 (m, 2H), 3.08–3.00 (m, 1H), 2.89–2.83 (m, 1H); ^13^C NMR (151 MHz, CD_3_OD) 172.6, 156.8, 143.8, 138.0, 133.6, 131.7 (q, *J* = 32.1 Hz), 130.9, 130.2, 129.8 (q, *J* = 32.6 Hz), 126.8 (q, *J* = 3.8 Hz), 126.2 (q, *J* = 3.7 Hz), 125.7 (q, *J* = 270.9 Hz), 125.6 (q, *J* = 271.2 Hz), 124.6 (q, *J* = 3.9 Hz). 51.0, 43.4, 40.6, 30.1; ESI-MS *m*/*z* 458.2 (M + H^+^); HRMS calcd for C_20_H_18_F_6_N_5_O^+^ (M + H^+^): 458.1410, found: 458.1407; [α]^20^_D_ = −6.1° (*c* = 0.1, MeOH).

### (*S*)-*N*-(1-(1*H*-Tetrazol-5-yl)-3-(4-(trifluoromethyl)phenyl)propan-2-yl)-2-(3-cyanophenyl)acetamide (**16j**) and (*S*)-*N*-(1-(1*H*-Tetrazol-5-yl)-3-(4-(trifluoromethyl)phenyl)propan-2-yl)-2-(3-(1*H*-tetrazol-5-yl)phenyl)acetamide (**16k**)

Step 1: **14** (30 mg, 0.11 mmol) was coupled with 2-(3-cyanophenyl)acetic acid (38 mg, 0.24 mmol) as described for **15a** to give 36 mg (84%) of **15j** as a white solid after flash chromatography (SiO_2_, EtOAc:Hept, 0:100 → 1:1): *R_f_* = 0.34 (EtOAc:Hept, 1:1); ^1^H NMR (400 MHz, CDCl_3_): δ 7.63–7.54 (m, 3H), 7.53–7.49 (m, 1H), 7.46–7.37 (m, 2H), 7.29–7.24 (m, 2H), 5.68 (s, 1H), 4.46–4.33 (m, 1H), 3.61–3.46 (m, 2H), 3.09–2.93 (m, 2H), 2.84–2.77 (m, 1H), 2.56–2.42 (m, 1H); ^13^C NMR (151 MHz, CDCl_3_): δ 169.7, 139.9, 135.7, 133.7, 132.8, 131.4, 130.1 (q, *J* = 32.6 Hz), 129.9, 129.4, 126.1 (q, *J* = 3.7 Hz), 124.0 (q, *J* = 272.2 Hz), 118.5, 116.9, 113.2, 47.3, 42.9, 38.9, 22.7; ESI-MS *m*/*z* 372.2 (M + H^+^); [α]^20^_D_ = −38.1° (*c* = 0.3, MeOH).

Step 2: **15j** (20 mg, 0.05 mmol) was reacted with NaN_3_ (14 mg, 0.22 mmol) as described for **16a** to give 4 mg (18%) of **16j** and 13 mg (52%) of **16 k** as white solids after purification by preparative HPLC. **16j**: *t_R_* = 9.52 min (purity 96.65% by HPLC at 220 nm); ^1^H NMR (600 MHz, CD_3_OD): δ 7.94–7.85 (m, 2H), 7.49–7.41 (m, 3H), 7.37–7.32 (m, 3H), 4.45–4.34 (m, 1H), 3.56 (s, 2H), 3.08–3.01 (m, 1H), 2.96–2.89 (m, 1H), 2.87–2.80 (m, 1H), 2.74–2.67 (m, 1H); ^13^C NMR (151 MHz, CD_3_OD): δ 173.1, 143.1, 138.2, 133.1, 130.9, 130.6, 130.0 (q, *J* = 32.1 Hz),129.0, 126.7, 126.3 (q, *J* = 3.8 Hz), 125.6 (q, *J* = 271.2 Hz), 49.6, 43.4, 40.0, 23.5; ESI-MS *m*/*z* 415.1 (M + H^+^); HRMS calcd for C_20_H_18_F_3_N_6_O^+^ (M + H^+^): 415.1488, found: 415.1486. **16k**: *t_R_* = 9.23 min (purity 99.30% by HPLC at 220 nm); ^1^H NMR (600 MHz, CD_3_OD): δ 7.86 (dt, *J* = 7.8, 1.4 Hz, 1H), 7.83–7.80 (m, 1H), 7.45–7.39 (m, 3H), 7.34 (d, *J* = 8.0 Hz, 2H), 7.19 (dt, *J* = 7.8, 1.5 Hz, 1H), 4.60–4.49 (m, 1H), 3.49–3.39 (m, 2H), 3.29–3.24 (m, 1H), 3.23–3.17 (m, 1H), 3.10–3.03 (m, 1H), 2.94–2.86 (m, 1H); ^13^C NMR (151 MHz, CD_3_OD): δ 172.9, 155.6, 143.7, 138.2, 132.7, 130.9, 130.5, 128.9, 126.6, 126.2 (q, *J* = 3.9 Hz), 50.9, 43.5, 40.7, 29.7; ESI-MS *m*/*z* 458.1 (M + H^+^); HRMS calcd for C_20_H_19_F_3_N_9_O^+^ (M + H^+^): 458.1659, found: 458.1656; [α]^20^_D_ = −25.0° (*c* = 0.2, MeOH).

### (*S*)*-N-(*1-(1*H*-Tetrazol-5-yl)-3-(4-(trifluoromethyl)phenyl)propan-2-yl)-2-(3-methoxyphenyl)acetamide (**16l**)

Step 1: **14** (22 mg, 0.08 mmol) was coupled with 2-(3-methoxyphenyl)acetic acid (29 mg, 0.17 mmol) as described for **15a** to give 20 mg (66%) of **15l** as a white solid after flash chromatography (SiO_2_, EtOAc:Hept, 1:2 → 1:1): *R_f_* = 0.38 (EtOAc:Hept, 1:1); ^1^H NMR (400 MHz, CD_3_OD): δ 7.50 (d, *J* = 8.0 Hz, 2H), 7.32 (d, *J* = 7.9 Hz, 2H), 7.15 (t, *J* = 7.9 Hz, 1H), 6.81–6.74 (m, 2H), 6.70 (d, *J* = 7.2 Hz, 1H), 4.43–4.31 (m, 1H), 3.75 (s, 3H), 3.41 (s, 2H), 3.07–2.98 (m, 1H), 2.97–2.87 (m, 1H), 2.86–2.76 (m, 1H), 2.73–2.63 (m, 1H); ^13^C NMR (151 MHz, CD_3_OD): δ 173.8, 161.3, 143.0, 137.9, 130.9130.0 (q, *J* = 32.2 Hz), 126.3 (q, *J* = 3.8 Hz), 130.5, 125.7 (q, *J* = 271.1 Hz), 122.2, 118.7, 115.7, 113.4, 55.6, 49.6, 43.8, 39.8, 23.4; ESI-MS *m*/*z* 377.1 (M + H^+^); [α]^20^_D_ = −21.6° (*c* = 0.2, MeOH).

Step 2: **15l** (18 mg, 0.05 mmol) was coupled with NaN_3_ (12 mg, 0.19 mmol) as described for **16a** to give 3.4 mg (17%) of **16l** as a white solid after purification by flash chromatography (SiO_2_, EtOAc:Hept, 2:3 then MeOH/DCM/AcOH, 5:95:0.1 →5:95:1): *t_R_* = 10.29 min (purity 98.21% by HPLC at 220 nm); ^1^H NMR (400 MHz, CD_3_OD): δ 7.47 (d, *J* = 8.0 Hz, 2H), 7.30 (d, *J* = 8.0 Hz, 2H), 7.11 (t, *J* = 7.9 Hz, 1H), 6.79–6.74 (m, 1H), 6.68–6.65 (m, 1H), 6.58 (d, *J* = 7.5 Hz, 1H), 4.62–4.44 (m, 1H), 3.74 (s, 3H), 3.32 (s, 1H), 3.29 (s, 1H), 3.25–3.12 (m, 2H), 3.07–2.98 (m, 1H), 2.93–2.83 (m, 1H); ^13^C NMR (151 MHz, CD_3_OD): δ 173.5, 161.2, 155.9, 143.7, 137.9, 130.9, 130.4, 129.8 (q, *J* = 32.1 Hz), 126.2 (q, *J* = 3.8 Hz), 125.8 (q, *J* = 272.4 Hz), 122.2, 115.7, 113.3, 55.6, 50.8, 43.9, 40.5, 29.7; ESI-MS *m*/*z* 420.2 (M + H^+^); HRMS calcd for C_20_H_21_F_3_N_5_O_2_^+^ (M + H^+^): 420.1641, found: 420.1640.

### (*S*)-*N*-(1-(1*H*-Tetrazol-5-yl)-3-(4-(trifluoromethyl)phenyl)propan-2-yl)-2-(3-ethoxyphenyl)acetamide (**16m**)

Step 1: K_2_CO_3_ (166 mg, 1.20 mmol), KI (20 mg, 0.12 mmol), and methyl 2-(3-hydroxyphenyl)acetate (83 μL, 0.60 mmol) in dry MeCN (3.60 mL) were added dropwise with ethyl bromide (178 μL, 2.40 mmol). The reaction mixture was stirred at rt for 24 h and, then at 55 °C overnight. After completion (TLC), the reaction mixture was cooled to rt, added water, and extracted with EtOAc (×3). The combined organic layers were washed with brine, dried over MgSO_4_, and concentrated in vacuo to give 102 mg (88%) of methyl 2-(3-ethoxyphenyl)acetate as oil that was used directly in the next step: *R*_*f*_ = 0.72 (EtOAc:Hept, 1:1); ^1^H NMR (400 MHz, CDCl_3_): δ 7.22 (t, *J* = 7.8 Hz, 1H), 6.88–6.74 (m, 3H), 4.03 (q, *J* = 7.0 Hz, 2H), 3.69 (s, 3H), 3.59 (s, 2H), 1.41 (t, *J* = 7.0 Hz, 3H); ^13^C NMR (101 MHz, CDCl_3_): δ 172.0, 159.2, 135.5, 129.6, 121.6, 115.6, 113.3, 63.5, 52.1, 41.4, 14.9. Spectra are in agreement with the literature reported data.^57^

Step 2: Methyl 2-(3-ethoxyphenyl)acetate (82 mg, 0.42 mmol) in THF (2.60 mL) was added 0.6 M LiOH_aq_ (2.10 mL), and the reaction mixture was stirred at rt for 45 min, then diluted with water, acidified with aqueous 1 M HCl_aq_, and extracted with EtOAc (×3). The organic layers were combined, washed with brine, dried over MgSO_4_, and concentrated in vacuo to give 76 mg (quantitative yield) of 2-(3-ethoxyphenyl)acetic acid as an off-white solid that was used directly in the next step: ^1^H NMR (400 MHz, CD_3_OD): δ 7.19 (t, *J* = 8.1 Hz, 1H), 6.98–6.72 (m, 3H), 4.02 (q, *J* = 7.0 Hz, 2H), 3.56 (s, 2H), 1.37 (t, *J* = 7.0 Hz, 3H); ^13^C NMR (101 MHz, CD_3_OD): δ 175.5, 160.5, 137.4, 130.4, 122.6, 116.6, 114.0, 64.4, 42.0, 15.2. Spectra are in agreement with the literature reported data.^57^

Step 3: **14** (31 mg, 0.12 mmol) was coupled with 2-(3-ethoxyphenyl)acetic acid (42 mg, 0.24 mmol) as described for **15a** to give 37 mg (84%) of **15m** as a white solid after purification by automated flash chromatography (SiO_2_, EtOAc:Hept, 0:100 → 1:1): *R_f_* = 0.20 (EtOAc:Hept, 1:1); ^1^H NMR (400 MHz, CD_3_OD): δ 7.50 (d, *J* = 8.0 Hz, 2H), 7.32 (d, *J* = 8.0 Hz, 2H), 7.14 (t, *J* = 7.8 Hz, 1H), 6.84–6.73 (m, 2H), 6.69 (dt, *J* = 7.6, 1.3 Hz, 1H), 4.42–4.31 (m, 1H), 3.99 (q, *J* = 7.0 Hz, 2H), 3.40 (s, 2H), 3.07–2.97 (m, 1H), 2.97–2.87 (m, 1H), 2.86–2.74 (m, 1H), 2.73–2.62 (m, 1H), 1.37 (t, *J* = 7.0 Hz, 3H); ESI-MS *m*/*z* 391.1 (M + H^+^); [α]^20^_D_ = −17.0° (*c* = 0.1, MeOH).

Step 4: **15m** (31 mg, 0.08 mmol) was reacted with NaN_3_ (22 mg, 0.33 mmol) as described for **16a** to give **16m** (20 mg, 58%) as an off-white solid after purification by flash chromatography (SiO_2_, DCM:MeOH, 95:5): *t_R_* = 10.76 min (purity 98.84% by HPLC at 220 nm); ^1^H NMR (600 MHz, CD_3_OD): δ 7.47 (d, *J* = 8.1 Hz, 2H), 7.29 (d, *J* = 8.0 Hz, 2H), 7.10 (t, *J* = 7.9 Hz, 1H), 6.75 (dd, *J* = 8.3, 2.6 Hz, 1H), 6.66 (t, *J* = 2.1 Hz, 1H), 6.57 (dt, *J* = 7.6, 1.2 Hz, 1H), 6.57 (d, *J* = 7.5 Hz, 1H), 4.55–4.48 (m, 1H), 3.97 (q, *J* = 7.0 Hz, 2H), 3.29–3.25 (m, 1H), 3.25–3.21 (m, 1H), 3.20–3.15 (m, 1H), 3.05–2.99 (m, 1H), 2.92–2.84 (m, 1H), 1.36 (t, *J* = 7.0 Hz, 3H); ^13^C NMR (151 MHz, CD_3_OD): δ 173.6, 160.5, 155.8, 143.7, 137.9, 130.9, 130.4, 129.8 (q, *J* = 32.2 Hz), 126.2 (q, *J* = 3.7 Hz), 125.8 (q, *J* = 271.1 Hz), 122.1, 116.3, 113.9, 64.3, 50.8, 43.9, 40.5, 29.7, 15.1; ESI-MS *m*/*z* 434.2 (M + H^+^); HRMS calcd for C_21_H_23_F_3_N_5_O_2_^+^ (M + H^+^): 434.1798, found: 434.1796; [α]^20^_D_ = −9.7° (*c* = 0.3, MeOH).

### (*S*)-*N*-(1-(1*H*-Tetrazol-5-yl)-3-(4-(trifluoromethyl)phenyl)propan-2-yl)-2-(3-(trifluoromethoxy)phenyl)acetamide (**16n**)

Step 1: **14** (29 mg, 0.11 mmol) was coupled with 2-(3-(trifluoromethoxy)phenyl)acetic acid (53 mg, 0.24 mmol) as described for **15a** to give **15n** (35 mg 74%) as a white solid after purification by automated flash chromatography (SiO_2_, EtOAc:Hept, 0:100 → 1:1): *R_f_* = 0.22 (EtOAc:Hept, 1:1); ^1^H NMR (400 MHz, CD_3_OD): δ 7.51 (d, *J* = 8.0 Hz, 2H), 7.39–7.28 (m, 3H), 7.17–7.05 (m, 3H), 4.45–4.33 (m, 1H), 3.48 (s, 2H), 3.09–3.00 (m, 1H), 2.98–2.87 (m, 1H), 2.87–2.77 (m, 1H), 2.74–2.63 (m, 1H); ^13^C NMR (101 MHz, CD_3_OD): δ 173.0, 150.5, 143.1, 139.2, 131.0, 130.8, 130.1 (q, *J* = 32.4 Hz), 128.8, 126.3 (q, *J* = 3.8 Hz), 125.7 (q, *J* = 271.1 Hz), 122.7, 121.9 (q, *J* = 255.5 Hz), 120.3, 118.6, 48.7, 43.2, 39.9, 23.5; ESI-MS *m*/*z* 431.1 (M + H^+^); [α]^20^_D_ = −24.3° (*c* = 0.3, MeOH).

Step 2: **15n** (22 mg, 0.05 mmol) was reacted with NaN_3_ (14 mg, 0.21 mmol) as described for **16a** to give 17 mg (69%) of **16n** as an off-white solid after purification by flash chromatography (SiO_2_, DCM:MeOH, 95:5 → 9:1 + 1% AcOH): *t_R_* = 11.40 min (purity 98.72% by HPLC at 220 nm); ^1^H NMR (600 MHz, CD_3_OD): δ 7.47 (d, *J* = 8.0 Hz, 2H), 7.33 (d, *J* = 7.9 Hz, 2H), 7.28 (t, *J* = 8.0 Hz, 1H), 7.13–7.08 (m, 1H), 7.02 (s, 1H), 7.00–6.95 (m, 1H), 4.61–4.49 (m, 1H), 3.42–3.33 (m, 2H), 3.27–3.14 (m, 2H), 3.07–3.01 (m, 1H), 2.91–2.84 (m, 1H); ^13^C NMR (151 MHz, CD_3_OD): δ 172.7, 156.2, 150.5, 143.8, 139.2, 131.0, 130.9, 129.9 (q, *J* = 32.2 Hz), 128.7, 126.2 (q, *J* = 3.9 Hz), 125.4 (q, *J* = 271.0 Hz), 122.7, 121.9 (q, *J* = 255.4 Hz), 120.2, 50.9, 43.3, 40.6, 29.9; ESI-MS *m*/*z* 474.1 (M + H^+^); HRMS calcd for C_20_H_18_F_6_N_5_O_2_^+^ (M + H^+^): 474.1359, found: 474.1364; [α]^20^_D_ = −8.6° (*c* = 0.2, MeOH).

### (*S*)-*N*-(1-(1*H*-Tetrazol-5-yl)-3-(4-(trifluoromethyl)phenyl)propan-2-yl)-2-cyclopentylacetamide (**16o**)

Step 1: **14** (30 mg, 0.11 mmol) was coupled with 2-cyclopentylacetic acid (62 mg, 0.24 mmol) as described for **15a** to give 30 mg (78%) of **15o** as a white solid after flash chromatography (SiO_2_, EtOAc:Hept, 0:100 → 1:1): *R_f_* = 0.31 (EtOAc:Hept, 1:1); ^1^H NMR (400 MHz, CDCl_3_): δ 7.60 (d, *J* = 8.2 Hz, 2H), 7.36 (d, *J* = 8.0 Hz, 2H), 5.63 (d, *J* = 8.0 Hz, 1H), 4.52–4.38 (m, 1H), 3.10–2.94 (m, 2H), 2.87–2.76 (m, 1H), 2.50–2.40 (m, 1H), 2.23–2.06 (m, 3H), 1.85–1.56 (m, 5H), 1.51–1.47 (m, 1H), 1.16–0.96 (m, 2H); ^13^C NMR (151 MHz, CDCl_3_): δ 173.0, 140.4, 130.0 (q, *J* = 32.6 Hz), 129.5, 126.1 (q, *J* = 3.9 Hz), 124.1 (q, *J* = 272.1 Hz), 117.2, 46.8, 42.9, 39.0, 37.2, 32.6, 32.5, 25.02, 24.99, 22.7; ESI-MS *m*/*z* 339.2 (M + H^+^); [α]^20^_D_ = −24.8° (*c* = 0.2, MeOH).

Step 2: **15o** (18 mg, 0.05 mmol) was reacted with NaN_3_ (14 mg, 0.21 mmol) as described for **16a** to give 2 mg (9%) of **16o** as a white solid after purification by flash chromatography (SiO_2_, EtOAc:MeOH, 100:0 → 80:20): *t_R_* = 10.69 min (purity 95.17% by HPLC at 220 nm); ^1^H NMR (600 MHz, CD_3_OD): δ 7.57 (d, *J* = 8.0 Hz, 2H), 7.44 (d, *J* = 8.0 Hz, 2H), 4.65–4.55 (m, 1H), 3.23–3.18 (m, 1H), 3.17–3.11 (m, 1H), 3.08–3.00 (m, 1H), 2.90–2.82 (m, 1H), 2.00–1.88 (m, 3H), 1.57–1.37 (m, 6H), 0.99–0.81 (m, 2H); ^13^C NMR (151 MHz, CD_3_OD): δ 175.3, 156.4, 144.1, 131.0, 129.9 (q, *J* = 32.1 Hz), 126.2 (q, *J* = 3.9 Hz), 125.8 (q, *J* = 269.8 Hz), 50.6, 43.1, 40.9, 38.5, 33.10, 33.05, 30.2, 25.71, 25.67; ESI-MS *m*/*z* 382.2 (M + H^+^); HRMS calcd for C_18_H_23_F_3_N_5_O^+^ (M + H^+^): 382.1849, found: 382.1847.

### (*S*)-*N*-(1-(1*H*-Tetrazol-5-yl)-3-(4-(trifluoromethyl)phenyl)propan-2-yl)hexanamide (**16p**)

Step 1: **14** (30 mg, 0.11 mmol) was coupled with hexanoic acid (27 mg, 0.24 mmol) as described for **15a** to give 31 mg (84%) of **15p** as a white solid after flash chromatography (SiO_2_, EtOAc:Hept, 0:100 → 1:1): *R_f_* = 0.35 (EtOAc:Hept, 1:1); ^1^H NMR (400 MHz, CDCl_3_): δ 7.60 (d, *J* = 8.2 Hz, 2H), 7.35 (d, *J* = 7.9 Hz, 2H), 5.66 (d, *J* = 7.8 Hz, 1H), 4.49–4.36 (m, 1H), 3.08 (dd, *J* = 14.0, 7.6 Hz, 1H), 2.99 (dd, *J* = 13.9, 7.9 Hz, 1H), 2.80 (dd, *J* = 16.9, 5.1 Hz, 1H), 2.46 (dd, *J* = 16.9, 4.1 Hz, 1H), 2.15 (td, *J* = 7.4, 3.3 Hz, 2H), 1.60–1.53 (m, 2H), 1.36–1.16 (m, 4H), 0.87 (t, *J* = 7.0 Hz, 3H); ^13^C NMR (151 MHz, CDCl_3_): δ 173.4, 140.3, 130.0 (q, *J* = 32.5 Hz), 129.5, 126.1 (q, *J* = 4.1 Hz), 124.1 (q, *J* = 272.1 Hz), 117.2, 46.9, 39.0, 36.7, 31.4, 25.3, 22.7, 22.4, 14.0; ESI-MS *m*/*z* 327.2 (M + H^+^); [α]^20^_D_ = −27.7° (*c* = 0.2, MeOH).

Step 2: **15p** (20 mg, 0.06 mmol) was reacted with NaN_3_ (17 mg, 0.25 mmol) as described for **16a** to give 21 mg (95%) of **16p** as a pale pink solid after trituration with heptane: *t_R_* = 10.65 min (purity 99.99% by HPLC at 220 nm); ^1^H NMR (600 MHz, CD_3_OD): δ 7.58 (d, *J* = 8.0 Hz, 2H), 7.44 (d, *J* = 8.0 Hz, 2H), 4.63–4.52 (m, 1H), 3.27–3.20 (m, 1H), 3.19–3.12 (m, 1H), 3.10–3.04 (m, 1H), 2.93–2.86 (m, 1H), 1.99 (td, *J* = 7.4, 1.1 Hz, 2H), 1.35 (p, *J* = 7.5 Hz, 2H), 1.20 (h, *J* = 7.4 Hz, 2H), 1.07–0.97 (m, 2H), 0.83 (t, *J* = 7.4 Hz, 3H); ^13^C NMR (151 MHz, CD_3_OD): δ 175.9, 155.4, 144.0, 131.0, 130.0 (q, *J* = 32.2 Hz), 126.3 (q, *J* = 3.8 Hz), 125.8 (q, *J* = 271.0 Hz), 50.4, 40.9, 37.0, 32.2, 29.8, 26.5, 23.3, 14.1; ESI-MS *m*/*z* 370.1 (M + H^+^); HRMS calcd for C_17_H_23_F_3_N_5_O^+^ (M + H^+^): 370.1849, found: 370.1848; [α]^20^_D_ = −10.0° (*c* = 0.3, MeOH).

### (*S*)*-N*-(1-(1*H*-Tetrazol-5-yl)-3-(4-(trifluoromethyl)phenyl)propan-2-yl)-3-phenylpropanamide (**16q**)

Step 1: **14** (30 mg, 0.11 mmol) was coupled with 3-phenylpropanoic acid (35 mg, 0.24 mmol) as described for **15a** to give 33 mg (81%) of **15q** as a white solid after flash chromatography (SiO_2_, EtOAc:Hept, 0:100 → 1:1): *R_f_* = 0.28 (EtOAc:Hept, 1:1); ^1^H NMR (400 MHz, CDCl_3_): δ 7.56 (d, *J* = 8.0 Hz, 2H), 7.31–7.18 (m, 5H), 7.16 (dd, *J* = 6.9, 1.8 Hz, 2H), 5.62 (d, *J* = 8.0 Hz, 1H), 4.43–4.30 (m, 1H), 3.05–2.82 (m, 4H), 2.72–2.61 (m, 1H), 2.55–2.43 (m, 2H), 2.41–2.31 (m, 1H); ^13^C NMR (151 MHz, CDCl_3_): δ 172.2, 140.4, 140.1, 129.9 (q, *J* = 32.5 Hz), 129.5, 128.8, 128.4, 126.7, 126.1 (q, *J* = 3.7 Hz), 124.1 (q, *J* = 272.2 Hz), 117.0, 46.8, 38.8, 38.3, 31.5, 22.5; ESI-MS *m*/*z* 361.2 (M + H^+^); [α]^20^_D_ = −22.7° (*c* = 0.1, MeOH).

Step 2: **15q** (20 mg, 0.06 mmol) was reacted with NaN_3_ (14 mg, 0.22 mmol) as described for **16a** to give 4 mg (17%) of **16q** as a white solid: *t_R_* = 10.73 min (purity 99.01% by HPLC at 220 nm); ^1^H NMR (600 MHz, CD_3_OD): δ 7.55 (d, *J* = 8.0 Hz, 2H), 7.36 (d, *J* = 7.9 Hz, 2H), 7.23–7.17 (m, 2H), 7.16–7.10 (m, 1H), 7.10–7.05 (m, 2H), 4.60–4.44 (m, 1H), 3.16 (dd, *J* = 14.9, 5.6 Hz, 1H), 3.08 (dd, *J* = 14.9, 8.0 Hz, 1H), 2.98 (dd, *J* = 13.9, 5.6 Hz, 1H), 2.85 (dd, *J* = 13.9, 8.7 Hz, 1H), 2.75–2.67 (m, 2H), 2.43–2.28 (m, 2H); ^13^C NMR (151 MHz, CD_3_OD): δ 174.9, 155.8, 143.9, 142.1, 131.0, 129.9 (q, *J* = 32.2 Hz), 129.4, 129.2, 127.2, 126.3 (q, *J* = 3.7 Hz), 125.8 (q, *J* = 271.0 Hz), 50.6, 40.7, 38.7, 32.6, 29.6; ESI-MS *m*/*z* 404.2 (M + H^+^); HRMS calcd for C_20_H_21_F_3_N_5_O^+^ (M + H^+^): 404.1692, found: 404.1693; [α]^20^_D_ = −6.1° (c = 0.1, MeOH).

### (*S*)-2-(2-((1-(1*H*-Tetrazol-5-yl)-3-(4-(trifluoromethyl)phenyl)propan-2-yl)amino)-2-oxoethyl)pyridin-1-ium 2,2,2-trifluoroacetate (**16r**)

Step 1: **14** (30 mg, 0.11 mmol) was coupled with 2-(pyridin-2-yl)acetic acid hydrochloride (41 mg, 0.24 mmol) as described for **15a** to give 33 mg (84%) of **15r** as a bright yellow solid after flash chromatography (SiO_2_, EtOAc:Hept, 0:100 → 100:0): *R_f_* = 0.18 (EtOAc); ^1^H NMR (400 MHz, CDCl_3_): δ 8.54–8.45 (m, 1H), 8.33–8.19 (m, 1H), 7.79–7.66 (m, 1H), 7.48 (d, *J* = 8.0 Hz, 2H), 7.30–7.20 (m, 3H), 4.48–4.33 (m, 1H), 3.81–3.64 (m, 2H), 3.13–2.95 (m, 2H), 2.78–2.67 (m, 1H), 2.57–2.42 (m, 1H); ^13^C NMR (101 MHz, CDCl_3_): δ 169.0, 154.6, 148.0, 140.5, 138.5, 129.6, 125.8 (q, *J* = 3.7 Hz), 124.7, 122.8, 117.1, 47.2, 44.3, 39.0, 22.8; ESI-MS *m*/*z* 348.2 (M + H^+^); [α]^20^_D_ = −31.1° (c = 0.1, MeOH).

Step 2: **15r** (20 mg, 0.05 mmol) was reacted with NaN_3_ (14 mg, 0.22 mmol) as described for **16a** to give 7 mg (23%) of **16r** as a white solid after purification by preparative HPLC: *t_R_* = 8.63 min (purity 98.70% by HPLC at 254 nm); ^1^H NMR (600 MHz, CD_3_OD): δ 8.66 (d, *J* = 5.8 Hz, 1H), 8.28 (td, *J* = 7.9, 1.7 Hz, 1H), 7.76 (td, *J* = 7.4, 5.6, 1.2 Hz, 1H), 7.62–7.53 (m, 3H), 7.44 (d, *J* = 8.0 Hz, 2H), 4.61–4.50 (m, 1H), 3.30–3.28 (m, 1H), 3.24–3.16 (m, 1H), 3.13–3.05 (m, 1H), 3.00–2.87 (m, 1H); ^13^C NMR (151 MHz, CD_3_OD): δ 167.7, 160.8 (q, *J* = 35.9 Hz), 153.9, 151.6, 143.8, 143.3, 142.3, 129.6, 128.6 (q, *J* = 32.1 Hz), 126.8, 125.0 (q, *J* = 3.8 Hz), 124.5, 124.3 (q, *J* = 271.1 Hz), 49.7, 48.2, 39.4, 27.9; ESI-MS *m*/*z* 392.1 (M + H^+^); HRMS calcd for C_18_H_18_F_3_N_6_O^+^ (M + H^+^): 391.1488, found: 391.1489; [α]^20^_D_ = −11.3° (c = 0.1, MeOH).

### (*S*)-3-(2-((1-(1*H*-Tetrazol-5-yl)-3-(4-(trifluoromethyl)phenyl)propan-2-yl)amino)-2-oxoethyl)pyridin-1-ium 2,2,2-trifluoroacetate (**16s**)

Step 1: **14** (30 mg, 0.11 mmol) was coupled with 2-(pyridin-3-yl)acetic acid (32 mg, 0.24 mmol) as described for **15a** to give 34 mg (86%) of **15s** as a white solid after flash chromatography (SiO_2_, EtOAc:Hept, 0:100 → 100:0): *R_f_* = 0.18 (EtOAc:Hept, 1:1); ^1^H NMR (400 MHz, CDCl_3_): δ 8.74 (s, 1H), 8.54 (d, *J* = 4.8 Hz, 1H), 7.77 (dt, *J* = 7.9, 1.9 Hz, 1H), 7.52 (d, *J* = 8.0 Hz, 2H), 7.40 (dd, *J* = 7.9, 5.0 Hz, 1H), 7.28 (d, *J* = 8.1 Hz, 2H), 6.81 (d, *J* = 7.9 Hz, 1H), 4.48–4.26 (m, 1H), 3.69–3.56 (m, 2H), 3.11–2.93 (m, 2H), 2.80–2.70 (m, 1H), 2.58–2.48 (m, 1H); ^13^C NMR (151 MHz, CDCl_3_): δ 169.6, 148.1, 146.1, 140.3, 139.5, 132.3, 129.9 (q, *J* = 32.8 Hz), 125.9 (q, *J* = 3.7 Hz), 129.5, 124.7, 124.1 (q, *J* = 272.2 Hz), 117.1, 47.5, 40.5, 39.0, 22.7; ESI-MS *m*/*z* 348.2 (M + H^+^).

Step 2: **15s** (20 mg, 0.06 mmol) was reacted with NaN_3_ (17 mg, 0.25 mmol) as described for **16a** to give 1.4 mg (5%) of **16s** as a white solid after purification by preparative HPLC: *t_R_* = 8.63 min (purity 97.08% by HPLC at 254 nm); ^1^H NMR (600 MHz, CD_3_OD): δ 8.62 (d, *J* = 5.3 Hz, 1H), 8.53 (s, 1H), 8.03 (dt, *J* = 8.0, 1.7 Hz, 1H), 7.76 (dd, *J* = 8.0, 5.5 Hz, 1H), 7.56 (d, *J* = 8.1 Hz, 2H), 7.42 (d, *J* = 8.0 Hz, 2H), 4.60–4.48 (m, 1H), 3.60–3.51 (m, 2H), 3.29–3.25 (m, 1H), 3.22–3.14 (m, 1H), 3.14–3.08 (m, 1H), 2.98–2.90 (m, 1H); ^13^C NMR (151 MHz, CD_3_OD): δ 171.0, 155.4, 145.5, 145.3, 143.8, 143.5, 136.3, 131.0, 130.0 (q, *J* = 32.4 Hz), 127.1, 126.3 (q, *J* = 3.7 Hz), 125.7 (q, *J* = 271.1 Hz), 51.0, 40.9, 39.9, 29.6; ESI-MS *m*/*z* 392.1 (M + H^+^); HRMS calcd for C_18_H_18_F_3_N_6_O^+^ (M + H^+^): 391.1488, found: 391.1487.

### (*S*)*-N*-(1-(1*H*-Tetrazol-5-yl)-3-(4-(trifluoromethyl)phenyl)propan-2-yl)-2-(pyridin-4-yl)acetamide (**16t**)

Step 1: **14** (30 mg, 0.11 mmol) was coupled with 4-(carboxymethyl)pyridin-1-ium chloride (41 mg, 0.24 mmol) as described for **15a** to give 33 mg (84%) of **15t** as a white solid after flash chromatography (SiO_2_, EtOAc:Hept, 0:100 → 100:0): *R_f_* = 0.18 (EtOAc:Hept, 1:1); ^1^H NMR (400 MHz, CDCl_3_): δ 8.72 (d, *J* = 7.9 Hz, 1H), 8.49 (d, *J* = 6.7 Hz, 2H), 7.95 (d, *J* = 6.3 Hz, 2H), 7.56 (d, *J* = 8.0 Hz, 2H), 7.40 (d, *J* = 8.0 Hz, 2H), 4.51–4.35 (m, 1H), 4.08 (d, *J* = 14.8 Hz, 1H), 3.88 (d, *J* = 14.7 Hz, 1H), 3.25–3.12 (m, 1H), 3.04–2.97 (m, 1H), 2.77–2.59 (m, 2H); ^13^C NMR (151 MHz, CDCl_3_): δ 167.4, 140.8, 140.5, 129.7, 127.9, 125.9 (q, *J* = 3.9 Hz), 117.7, 48.2, 43.3, 39.4, 22.5; ESI-MS *m*/*z* 348.2 (M + H^+^).

Step 2: **15t** (20 mg, 0.06 mmol) was reacted with NaN_3_ (17 mg, 0.26 mmol) as described for **16a**. The aqueous phase was neutralized with 10% aq. NaOH and extracted with EtOAc (×3), and the combined organic layers were washed with brine, dried over MgSO_4_, and concentrated in vacuo to give 17 mg (78%) of **16t** as a white solid: *t_R_* = 8.30 min (purity 98.48% by HPLC at 254 nm); ^1^H NMR (400 MHz, CD_3_OD): δ 8.25 (s, 2H), 7.42 (d, *J* = 8.0 Hz, 2H), 7.28 (d, *J* = 8.0 Hz, 2H), 6.98 (d, *J* = 5.8 Hz, 2H), 4.53–4.43 (m, 1H), 3.30 (s, 2H), 3.19–3.13 (m, 1H), 3.13–3.05 (m, 1H), 3.03–2.96 (m, 1H), 2.84–2.76 (m, 1H); ^13^C NMR (151 MHz, CD_3_OD): δ 171.4, 155.7, 149.4, 147.8, 143.8, 131.0, 129.9 (q, *J* = 32.2 Hz), 126.3 (q, *J* = 3.8 Hz), 126.0, 125.8 (q, *J* = 271.0 Hz), 50.9, 49.6, 40.8, 29.8; ESI-MS *m*/*z* 391.1 (M + H^+^); HRMS calcd for C_18_H_18_F_3_N_6_O^+^ (M + H^+^): 391.1488, found: 391.1488; [α]^20^_D_ = −9.1° (*c* = 0.3, MeOH).

### (*S*)*-N*-(1-(1*H*-Tetrazol-5-yl)-3-(4-(trifluoromethyl)phenyl)propan-2-yl)-2-(1H-imidazol-5-yl)acetamide 2,2,2-trifluoroacetate (**16u**)

Step 1: **14** (30 mg, 0.11 mmol) was coupled with 2-(1*H*-imidazol-5-yl)acetic acid hydrochloride (38 mg, 0.24 mmol) as described for **15a** to give 11 mg (29%) of **15u** as colorless oil after flash chromatography (SiO_2_, DCM:MeOH, 100:0 → 9:1): *R_f_* = 0.54 (DCM:MeOH, 9:1); ^1^H NMR (400 MHz, CD_3_OD): δ 7.78 (s, 1H), 7.56 (d, *J* = 8.0 Hz, 2H), 7.39 (d, *J* = 7.9 Hz, 2H), 6.93 (s, 1H), 4.45–4.33 (m, 1H), 3.49 (s, 2H), 3.08–3.00 (m, 1H), 3.00–2.90 (m, 1H), 2.87–2.76 (m, 1H), 2.73–2.61 (m, 1H); ^13^C NMR (151 MHz, CD_3_OD): δ 172.4, 143.1, 136.1, 132.4, 130.9, 130.1 (q, *J* = 32.2 Hz), 126.4 (q, *J* = 3.8 Hz), 125.7 (q, *J* = 271.1 Hz), 118.6, 118.0, 61.5, 40.0, 34.9, 23.3; ESI-MS *m*/*z* 337.2 (M + H^+^).

Step 2: **15u** (11 mg, 0.03 mmol) was reacted with NaN_3_ (9 mg, 0.13 mmol) as described for **16a** to give 1.7 mg (10%) of **16u** as a white solid after purification by preparative HPLC: *t_R_* = 8.30 min (purity 97.99% by HPLC at 220 nm); ^1^H NMR (600 MHz, CD_3_OD): δ 8.76 (s, 1H), 7.58 (d, *J* = 8.0 Hz, 2H), 7.44 (d, *J* = 7.9 Hz, 2H), 7.23 (s, 1H), 4.56–4.50 (m, 1H), 3.61–3.46 (m, 2H), 3.27 (dd, *J* = 14.9, 4.7 Hz, 1H), 3.17 (dd, *J* = 14.8, 8.6 Hz, 1H), 3.09 (dd, *J* = 13.9, 5.6 Hz, 1H), 2.94 (dd, *J* = 13.9, 9.1 Hz, 1H); ^13^C NMR (151 MHz, CD_3_OD): δ 169.5, 143.7, 134.9, 131.0, 130.0 (q, *J* = 32.2 Hz), 129.1, 126.4 (q, *J* = 3.9 Hz), 125.8 (q, *J* = 270.5 Hz), 118.3, 51.1, 40.9, 32.0, 29.4; ESI-MS *m*/*z* 380.2 (M + H^+^); HRMS calcd for C_16_H_17_F_3_N_7_O^+^ (M + H^+^): 380.1441, found: 380.1441.

### (*S*)-5-(2-((1-(1*H*-Tetrazol-5-yl)-3-(4-(trifluoromethyl)phenyl)propan-2-yl)amino)-2-oxoethyl)-2-chloropyridin-1-ium 2,2,2-trifluoroacetate (**16v**)

Step 1: **14** (29 mg, 0.11 mmol) was coupled with 2-(6-chloropyridin-3-yl)acetic acid (42 mg, 0.24 mmol) as described for **15a** to give 28 mg (65%) of **15v** as a white solid after purification by automated flash chromatography (SiO_2_, EtOAc:Hept, 1:9 → 1:1): *R_f_* = 0.16 (EtOAc:Hept, 1:2); ^1^H NMR (400 MHz, CD_3_OD): δ 8.19 (d, *J* = 2.5 Hz, 1H), 7.60–7.50 (m, 3H), 7.37 (d, *J* = 8.0 Hz, 2H), 7.31 (d, *J* = 8.2 Hz, 1H), 4.46–4.35 (m, 1H), 3.47 (s, 2H), 3.11–3.00 (m, 1H), 2.97–2.86 (m, 1H), 2.88–2.78 (m, 1H), 2.73–2.61 (m, 1H); ^13^C NMR (151 MHz, CD_3_OD): δ 172.2, 150.9, 150.8, 143.1, 141.3, 132.2, 130.9, 130.2 (q, *J* = 32.2 Hz), 126.4 (q, *J* = 4.0 Hz), 125.7 (q, *J* = 271.1 Hz), 125.3, 118.6, 49.6, 40.1, 39.7, 23.5; ESI-MS *m*/*z* 382.4 (M + H^+^); [α]^20^_D_ = −24.4° (*c* = 0.1, MeOH).

Step 2: **15v** (23 mg, 0.06 mmol) was reacted with NaN_3_ (18 mg, 0.28 mmol) as described for **16a** to give **16v** (6.7 mg, 21%) as a white solid after purification by preparative HPLC: *t_R_* = 9.49 min (purity 99.17% by HPLC at 220 nm); ^1^H NMR (600 MHz, CD_3_OD): δ 8.12–8.08 (m, 1H), 7.52 (d, *J* = 8.1 Hz, 2H), 7.40 (dd, *J* = 8.2, 2.5 Hz, 1H), 7.37 (d, *J* = 8.0 Hz, 2H), 7.28 (dd, *J* = 8.3, 0.7 Hz, 1H), 4.57–4.50 (m, 1H), 3.35 (s, 2H), 3.30–3.23 (m, 2H), 3.22–3.15 (m, 1H), 3.11–3.05 (m, 1H), 2.93–2.86 (m, 1H); ^13^C NMR (151 MHz, CD_3_OD): δ 172.0, 150.8, 143.7, 141.2, 132.1, 130.9, 130.0 (q, *J* = 32.2 Hz), 126.3 (q, *J* = 3.8 Hz), 125.7 (q, *J* = 271.0 Hz), 125.2, 50.9, 40.8, 39.7, 29.6; ESI-MS *m*/*z* 425.5 (M + H^+^); HRMS calcd for C_18_H_17_ClF_3_N_6_O^+^ (M + H^+^): 425.1099, found: 425.1099; [α]^20^_D_ = −6.5° (c = 0.1, MeOH).

### (*S*)-3-(2-((1-(1*H*-Tetrazol-5-yl)-3-(4-(trifluoromethyl)phenyl)propan-2-yl)amino)-2-oxoethyl)-5-bromopyridin-1-ium 2,2,2-trifluoroacetate (**16w**)

Step 1: **14** (29 mg, 0.11 mmol) was reacted with 2-(5-bromopyridin-3-yl)acetic acid (51 mg, 0.24 mmol) as described for **15a** to give 28 mg (60%) of **15w** as a white solid after purification by automated flash chromatography (SiO_2_, EtOAc:Hept, 0:100 → 75:25): *R_f_* = 0.25 (EtOAc); ^1^H NMR (400 MHz, CD_3_OD): δ 8.51 (d, *J* = 2.2 Hz, 1H), 8.34 (d, *J* = 1.9 Hz, 1H), 7.85 (t, *J* = 2.1 Hz, 1H), 7.53 (d, *J* = 8.0 Hz, 2H), 7.37 (d, *J* = 8.0 Hz, 2H), 4.46–4.34 (m, 1H), 3.57–3.41 (m, 2H), 3.09–3.00 (m, 1H), 2.98–2.88 (m, 1H), 2.88–2.77 (m, 1H), 2.74–2.63 (m, 1H); ^13^C NMR (151 MHz, CD_3_OD): δ 172.0, 149.8, 149.1, 143.0, 141.1, 135.0, 130.8, 130.1 (q, *J* = 32.3 Hz), 126.4 (q, *J* = 3.8 Hz), 125.7 (q, *J* = 271.1 Hz) 121.6, 118.6, 48.7, 39.99, 39.98, 23.5; ESI-MS *m*/*z* 426.0 (M + H^+^).

Step 2: **15w** (28 mg, 0.06 mmol) was reacted with NaN_3_ (17 mg, 0.26 mmol) as described for **16a** to give **16w** (17 mg, 46%) as a white solid after purification by preparative HPLC: *t_R_* = 8.83 min (purity 97.40% by HPLC at 254 nm); ^1^H NMR (600 MHz, CD_3_OD): δ 8.57 (s, 1H), 8.32 (s, 1H), 7.88 (t, *J* = 2.0 Hz, 1H), 7.52 (d, *J* = 8.2 Hz, 2H), 7.38 (d, *J* = 7.9 Hz, 2H), 4.56–4.45 (m, 1H), 3.42 (s, 2H), 3.30–3.22 (m, 1H), 3.22–3.15 (m, 1H), 3.11–3.05 (m, 1H), 2.95–2.88 (m, 1H); ^13^C NMR (151 MHz, CD_3_OD): δ 171.4, 160.6 (q, *J* = 39.1 Hz),155.4, 148.7, 148.0, 143.6, 142.6, 135.6, 130.9, 130.0 (q, *J* = 32.1 Hz), 126.3 (q, *J* = 3.9 Hz), 125.7 (q, *J* = 271.1 Hz), 121.8, 116.9 (q, *J* = 287.3 Hz) 50.9, 40.7, 39.9, 29.6; ESI-MS *m*/*z* 469.1 (M + H^+^); HRMS calcd for C_18_H_17_BrF_3_N_6_O^+^ (M + H^+^): 469.0593, found: 469.0601; [α]^20^_D_ = −6.5° (c = 0.3, MeOH).

### In Vitro Pharmacology

#### Cell Maintenance

The hFFA2-eYFP and hFFA3-eYFP Flp-In T-Rex 293 cell lines were maintained in Dulbecco’s modified Eagle mMedium (DMEM, ThermoFisher #11965092) supplemented with 10% (v/v) fetal bovine serum, 100 U/mL penicillin, 100 μg/mL streptomycin, 5 μg/mL blasticidin S, and 200 μg/mL hygromycin B and incubated at 37 °C and 5% CO_2_ in a humidified incubator. Cells were passaged at 70–80% confluency.

#### [^35^S]GTPγS Assay

Functional activity of the compounds were tested in a [^35^S]GTPγS incorporation assay using membranes of Flp-In T-Rex 293 cells induced to express human FFA2-eYFP. To assess inhibition of agonist stimulation, 5 μg of membrane preparations were pre-incubated with antagonist compounds in the assay buffer (20 mM HEPES, 5 mM MgCl_2_, 160 mM NaCl, 0.05% fatty-acid-free bovine serum albumin; pH 7.5) for 15 min at room temperature prior to addition of agonist. The reaction was initiated by addition of [^35^S]GTPγS (100 nCi per reaction) containing 1 μM GDP and incubated at 30 °C for 60 min. The reaction was terminated by rapid vacuum filtration through GF/C glass fiber filter-bottom 96-well microplates (PerkinElmer Life Sciences, Beaconsfield, UK) using a UniFilter FilterMate Harvester (PerkinElmer). Unbound radioligands were removed from filters by three washes with ice-cold PBS. MicroScint-20 (PerkinElmer) was added to dried filters, and [^35^S]GTPγS binding was quantified by liquid scintillation spectroscopy.

#### cAMP Assay

To determine the compound’s ability to activate or inhibit the G_i_-coupled FFA2 or FFA3, intracellular cAMP concentrations were determined using Flp-In T-Rex293 cells induced to express hFFA2-eYFP or hFFA3-eYFP. Experiments were carried out using a homogeneous time-resolved FRET-based detection kit (CisBio, PerkinElmer #62AM9PEC) according to the manufacturer’s protocol. The day before the assay, 10,000 cells/well were plated in DMEM supplemented with penicillin/streptomycin (100 units/mL and 100 mg/mL, respectively), 5 μg/mL blasticidin S, and 200 μg/mL hygromycin B in 96-well cell culture microplates (Greiner Bio-One) and induced to express hFFA2-eYFP or hFFA3-YFP with 100 ng/mL doxycycline.

In agonist mode, the next day, the agonist was added in Hank’s balanced salt solution supplemented with 20 mM HEPES, 1 mM MgCl_2_, and 1 mM CaCl_2_ at pH 7.4 (HBSS) together with 50 μM 3-isobutyl-1-methylxanthine (IBMX) and 3 μM forskolin. After 25 min pf incubation at rt, agonist-induced intracellular cAMP production was assessed by stopping the reactions and measuring the output with an Envision plate reader using a time-resolved fluorescence protocol, measuring the light emission at 620 and 665 nm.

In antagonist mode, the cells were pre-incubated for 20 min with antagonist test compounds in HBSS together with 50 μM IBMX at rt. Then, the cells were further incubated for 25 min at rt in HBSS with the agonist compound, sodium propionate at EC_80_ (51.4 μM for FFA2, 13.2 μM for FFA3), and 3 μM forskolin. Reactions were stopped, and the output was measured with an Envision plate reader using a time-resolved fluorescence protocol, measuring the light emission at 620 and 665 nm. All analyses were performed in triplicate and analyzed using GraphPad Prism (version 9).

#### Kinetic Aqueous Solubility

Twenty microliters of a 10 mM compound stock solution in DMSO was added to an Eppendorf tube containing 980 μL of phosphate buffer (10 mM, pH = 7.4). The samples were incubated in an Eppendorf Thermomixer (25 °C, 800 rpm) for 24 h. Afterward, the samples were centrifuged for 5 min at 11,000 rpm and the supernatant was filtered (0.2 μm PTFE membrane) before analysis by HPLC. Each compound was analyzed in duplicate. The solubility was calculated from the peak area relative to the reference samples (200 μM in MeOH/MilliQ H_2_O, 60/40, v/v).

#### Chemical Stability

Duplicates of 1 mL of a 50 μM solution were prepared by adding the 10 mM stock solution to a 1.5 mL Eppendorf tube and diluting it with PBS_7.4_. The Eppendorf tubes were incubated at 37 °C with gentle shaking (650 rpm) using an Eppendorf Thermomixer. The samples were briefly vortexed, and 50 μL aliquots were withdrawn at the time points 0, 12, 48, etc.; added to 250 μL vials; and analyzed immediately by HPLC. The chemical stability in PBS_7.4_ was determined at every time point in percentage relative to the 0 h time point.

#### Lipophilicity (logD_7.4_)

The assay was performed in duplicates essentially as previously described.^58^ A glass vial with a screw cap (8 mL) was charged with the test compound (40 μL, 10 mM in DMSO), PBS_7.4_ (0.01 M, 1980 μL), and 1-octanol (1980 μL). The vial was capped and sealed with a Parafilm and shaken at 700 rpm using an IKA KS 125 basic shaker for 24 h at 25 °C. The Parafilm was removed, and the sample was allowed to equilibrate for 1 h before analysis. One hundred microliters of the octanol phase was withdrawn and diluted 1:10 with MeOH (+0.1% TFA)/MilliQ water (4:1, v/v) and analyzed by HPLC. The interface was removed, and the PBS_7.4_ phase analyzed directly by HPLC. All analysis was performed in duplicates, and log D_7.4_ values were calculated from the peak areas (mAU·min) and adjusted for difference in injection volume and concentration-absorption effects from the solvents, using two calibration points per compound per solvent, and dilution of the octanol phase.

#### Microsomal Stability

The assay was performed in duplicates essentially as described previously,^58^ using male CD1 mouse liver microsomes (Thermo Fischer Scientific, 20 mg/mL), NADPH-regenerating agent Solution A and Solution B (Promega), potassium phosphate buffer (100 mM, pH 7.4), and test compounds (1 mM, diluted from 10 mM DMSO stock in the buffer). The samples were analyzed in an Agilent 6130 Mass Spectrometer instrument using electron spray ionization (ESI) coupled to an Agilent 1200 HPLC system (ESI-LCMS) with a C18 reverse phase column (Zorbax Eclipse XBD-C18, 4.6 mm × 50 mm) using a linear gradient of the binary solvent system of 100% mobile phase A (water:MeCN:formic acid, 95:5:0.1 v/v%) to 100% mobile phase B (MeCN:formic acid, 100:0.1 v/v %) in 6 min, with a flow rate of 1 mL/min.

To a pre-warmed solution of potassium phosphate buffer (135.6 μL) in an Eppendorf tube at 37 °C were added Solution A (7.5 μL), Solution B (1.5 μL), microsomes (3.9 μL), and the test compound (1 mM, 1.5 μL, final concentration = 10 μM). The tube was briefly vortexed and incubated at 37 °C in a thermomixer. At 0, 15, 30, 45, and 60 min, 25 μL of each sample was transferred to an ice-cold solution containing an internal standard (5 μM in MeCN, 12.5 μL). The sample was briefly vortexed and centrifuged for 5 min at 10.000*g.* The supernatant (25 μL) was transferred to HPLC vial and analyzed by ESI-LCMS in single ion mode. The data was analyzed using GraphPad Prism (version 9).

#### Human Neutrophil Isolation

Human peripheral blood neutrophils were isolated from healthy volunteer buffy coats. Initially, the majority of red blood cells were sedimented using 2% w/v dextran sulfate in a 0.9% NaCl solution for 15 min. Next, the upper phase was centrifuged for 10 min at 200*g* and the supernatant was discarded. The pellet was resuspended in 0.9% NaCl solution, and neutrophils were separated from here using Lymphoprep (STEMCELL Technologies #07851) and density-gradient centrifugation (400*g* for 30 min). Then, the remaining red blood cells were subjected to hypotonic lysis by addition of 10 mL of sterile water followed by addition of 10 mL of 1.8% NaCl solution. Cell count and viability were determined using trypan blue and a Countess cell counter. Isolated neutrophils were characterized by May–Grunwald–Giemsa staining and immediately used for migration assays or assessment of respiratory burst.

#### Human Neutrophil Migration

Freshly isolated human peripheral neutrophils were used in migration assays using the Boyden style Corning HTS Transwell 96 well system with 3 μm pores (Corning #3385). Initially, neutrophils were pre-incubated for 30 min with shown concentrations of compounds or vehicle (DMSO) in the RPMI 1640 medium supplemented with 10 mM HEPES (chemotaxis buffer) in a humidified atmosphere at 37 °C and 5% CO_2._ Next, 135,000 cells in the chemotaxis buffer with a vehicle or compound were added to the filter inserts. To the bottom chamber, chemotaxis buffer supplemented with compounds or a vehicle and 1 mM of the agonist sodium propionate or vehicle was added. The plate was incubated for 1 h in a humidified atmosphere at 37 °C and 5% CO_2_. Finally, the number of cells that had migrated to the bottom chamber was assessed using the ATPLite Luminescence assay system (PerkinElmer #6016943) according to the manufacturer’s instructions. Luminescence was measured on an LUMIstar Omega microplate reader (PerkinElmer). Experiments were performed in triplicate and analyzed using GraphPad Prism (version 9). Fold response was calculated as the relative migration of samples compared to the vehicle control.

#### Human Neutrophil Respiratory Burst

Human neutrophil respiratory burst was assessed via luminol-amplified chemiluminescence. Freshly isolated human neutrophils were seeded in a concentration of 100,000 cells/well in a flat bottom, white 96-well plate (PerkinElmer cat #6005680). Cells were pre-incubated for 30 min with test compounds or vehicles (DMSO) in HBSS in a humidified atmosphere at 37 °C and 5% CO_2._ Next, luminol (Merck cat #521-31-3) was added to a final concentration of 10 μM. Lastly, the vehicle or the FFA2 agonist, sodium propionate, was added to a final concentration of 1 mM. Immediately thereafter, chemiluminescence was evaluated with an LUMIstar Omega microplate reader (PerkinElmer) where light emission was recorded for 1 s/well every 60 s for 1 h at 37 °C. Experiments were performed in triplicate and analyzed using GraphPad Prism (version 9). Fold response was calculated as the relative luminescence of samples compared to the vehicle control.

### Pharmacokinetic Study

The study was performed by Bienta (www.bienta.net). Study design, animal selection, handling, and treatment were all in accordance with the Enamine PK study protocols and Institutional Animal Care and Use Guidelines. Animal treatment and plasma sample preparation were conducted by the Animal Laboratory personnel at Enamine/Bienta. Male BALB/cAnC mice (10–11 weeks old, body weight: 16.0–21.7 g and average body weight across all groups: 19.1 g, SD = 1.2 g) were used in this study. The animals were randomly assigned to the treatment groups before the pharmacokinetic study; all animals were fasted for 4 h before dosing. Intravenous (IV) and peroral (PO) routes of administration were done according to the following; eight time points for each route (3, 7, 15, 30, 60, 120, 240, and 480 min) for the IV route and (5, 10, 15, 30, 60, 120, 240, and 480 min) for the PO route were set for this pharmacokinetic study. Each of the time point treatment group included three animals. There was also one control animal. Compound **16l** in the vehicle (DMSO–Cremophor EL–water w/5% mannitol, 1:1:8) was dosed at 10 mg/kg po and 5 mg/kg iv. Dosing volumes of compounds or vehicle were 5 mL/kg. Mice were injected iv with 2,2,2-tribromoethanol at a dose of 150 mg/kg prior to drawing the blood. Blood collection was performed from the orbital sinus in microtainers containing K_3_EDTA. Animals were sacrificed by cervical dislocation after the blood sample collection. Blood samples were centrifuged for 10 min at 3000 rpm. Before analysis, plasma samples (40 μL) were added 200 μL of a solution with transilast as the internal standard (200 ng/mL in water–methanol mixture 1:9, v/v). After mixing by pipetting and centrifuging for 4 min at 6000 rpm, 0.5 μL of each supernatant was analyzed by HPLC-MS/MS on an API 3000 PE instrument.

## Supporting Information Available

LCMS traces for representative compounds, microsomal stability of selected compounds, effect on fMLF-induced neutrophil migration and respiratory burst, effect of **16l** in cells not expressing FFA2, and analysis of enantiomeric purity (PDF)
Molecular formula strings (CSV)

## Abbreviations

DIAD: diisopropyl azodicarboxylate
DIPEA: diisopropylethylamine
DMEM: Dulbecco’s modified Eagle medium
EDC: 1-ethyl-3-(3-dimethylaminopropyl)carbodiimide
eYFP: enhanced yellow fluorescent protein
FFA2: free fatty acid receptor 2 (GPR43)
FFA3: free fatty acid receptor 3 (GPR41)
fMLP: *N*-formyl-Met-Leu-Phe (fMLF)
GLP-1: glucagon-like peptide-1
HEPES: 4-(2-hydroxyethyl)-1-piperazineethanesulfonic acid
Hept: *n*-heptane
HOBt: hydroxybenzotriazole
HBSS: Hank’s balanced salt solution
IBCF: isobutyl chloroformate
IBMX: 3-isobutyl-1-methylxanthine
Imi: imipramine
LLE: ligand lipophilicity efficiency
NMM: *N-*methylmorpholine
PE: petroleum ether (pb 60–80 °C)
PTFE: polytetrafluoroethylene
PyBOP: benzotriazole-1-yloxytripyrrolidinophosphonium hexafluorophosphate
SCFA: short-chain fatty acid
