# Supplementary material for: Discovery of Potent Tetrazole Free Fatty Acid Receptor 2 Antagonists
Source: J Med Chem. 2023 May 2;66(9):6105–21. doi: 10.1021/acs.jmedchem.2c01935 (PMC10547238; doi:10.1021/acs.jmedchem.2c01935)
Supplement: Supplementary file 1 — jm2c01935_si_001.pdf [file jm2c01935_si_001.pdf]

Supporting Information

Discovery of Potent Tetrazole Free Fatty Acid Receptor 2 Antagonists

Alice Valentini[†], *Katrine Schultz-Knudsen*[†], *Anders Højgaard Hansen*[§], *Argyro Tsakoumagkou*[†], *Laura Jenkins*[‡], *Henriette B. Christensen*[§], *Asmita Manandhar*[†], *Graeme Milligan*[‡], *Trond Ulven*[†§]*, *Elisabeth Rexen Ulven*[†]*

[†]Department of Drug Design and Pharmacology, University of Copenhagen, Universitetsparken 2, DK-2100 Copenhagen, Denmark; [‡]Centre for Translational Pharmacology, School of Molecular Biosciences, College of Medical, Veterinary and Life Sciences, University of Glasgow, Glasgow G12 8QQ, Scotland, United Kingdom; [§]Department of Physics, Chemistry and Pharmacy, University of Southern Denmark, Campusvej 55, DK-5230 Odense M, Denmark

*E-mail: eru@sund.ku.dk, tu@sund.ku.dk

Table of Contents

Microsomal stability of selected compounds ______ S2
Effect on fMLF-induced neutrophil migration and respiratory burst ______ S3
Effect of 16l in cells not expressing FFA2 ______ S4
Analysis of enantiomeric purity ______ S5
HPLC chromatograms for **16l** (TUG-2304) ______ S6
HPLC chromatograms for **16w** ______ S7
References ______ S8

Microsomal stability of selected compounds

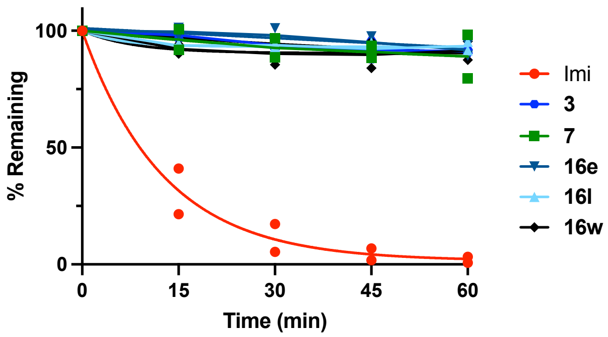

Figure S1. Microsomal stability of selected compounds and imipramine (Imi) (n = 2, individual data points are shown).

Effect on fMLP-induced neutrophil migration and respiratory burst

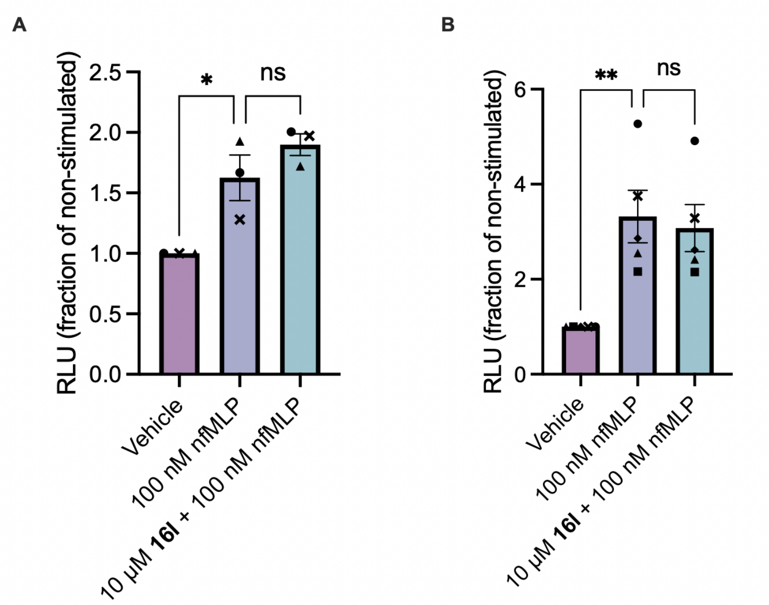

Figure S2. Effect of **16l** on *N*-formyl peptide (fMLP)-induced (A) neutrophil migration and (B) respiratory burst (bars represent mean ± SEM from ≥3 independent experiments, each performed in triplicate). *, $p < 0.05$; **, $p < 0.005$; ns, not significant.

Effect of 16l in cells not expressing FFA2

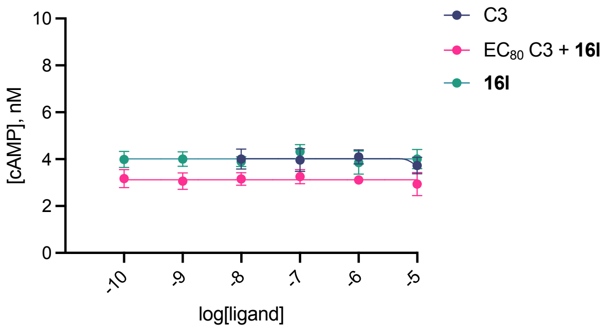

Figure S3. Concentration-response curves of the effect of propionate (C3), **16l**, and **16l** in the presence of 51.4 μM C3 (EC_{80}) on non-induced hFFA2-eYFP Flp-In T-Rex 293 cells (bars represent mean ± SEM from ≥3 independent experiments, each performed in triplicate).

Analysis of enantiomeric purity

For commercial compounds 4-(trifluoromethyl)-DL-phenylalanine (BLDpharm) and 4-(trifluoromethyl)-L-phenylalanine (**9**) (Fluorochem): Reverse phase chiral HPLC was carried out essentially as previously reported[1] on a Dionex HPLC system (Thermo Scientific) using a Crownpak-CR-(-) column (150 mm × 4 mm, Daicel 28714); isocratic elution aq. $HClO_4$ (pH 1)/MeOH 90:10, flow rate 1 mL/min, temperature 40 °C, detection wavelength 210 nm. Retention times: 4-(trifluoromethyl)-DL-phenylalanine 14.18 min and 17.90 min; 4-(trifluoromethyl)-L-phenylalanine (**9**) 14.00 min.

For *tert*-butyl (*R*/*S*)-(1-hydroxy-3-(4-(trifluoromethyl)-phenyl)propan-2-yl)carbamate and *tert*-butyl (*S*)-(1-hydroxy-3-(4-(trifluoromethyl)phenyl)-propan-2-yl)carbamate (**11**): Normal phase chiral HPLC was carried out on a Dionex HPLC system (Thermo Scientific) using a CHIRALPAK® column AD-H (4.6 mm × 250 mm, Daicel 19325); isocratic elution with n-heptane/isopropanol 90:10, flow rate 0.5 mL/min, detection wavelength 210 nm. Retention times: *tert*-butyl (*R*/*S*)-(1-hydroxy-3-(4-(trifluoromethyl)-phenyl)propan-2-yl)carbamate 15.81 min and 18.15 min; *tert*-butyl (*S*)-(1-hydroxy-3-(4-(trifluoromethyl)phenyl)-propan-2-yl)carbamate (**11**) 15.75 min.

Data were acquired and processed using Chromeleon 7.

Chiral HPLC chromatograms of 4-(trifluoromethyl)-DL-phenylalanine and 4-(trifluoromethyl)-L-phenylalanine (**9**)

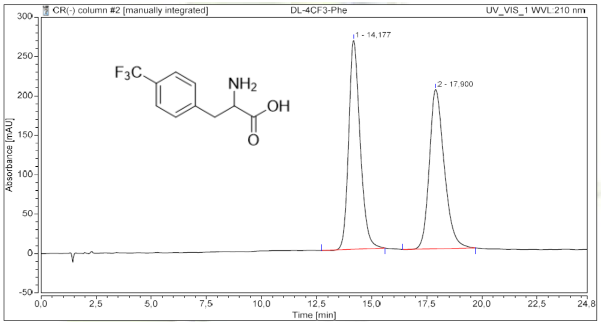

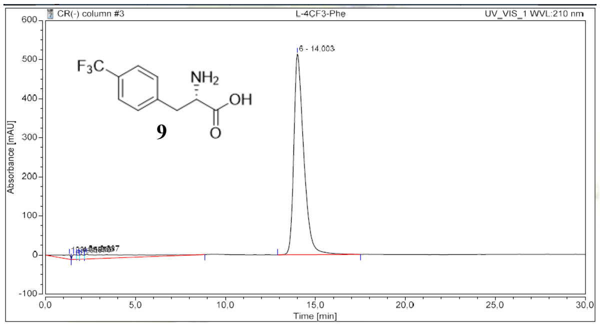

Chiral HPLC chromatograms of compounds racemic *tert*-butyl (1-hydroxy-3-(4-(trifluoromethyl)-phenyl)propan-2-yl)carbamate and intermediate **11**.

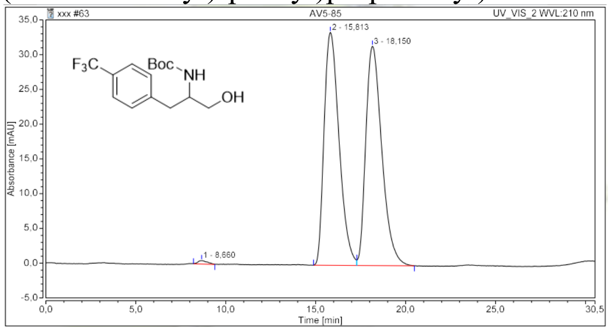

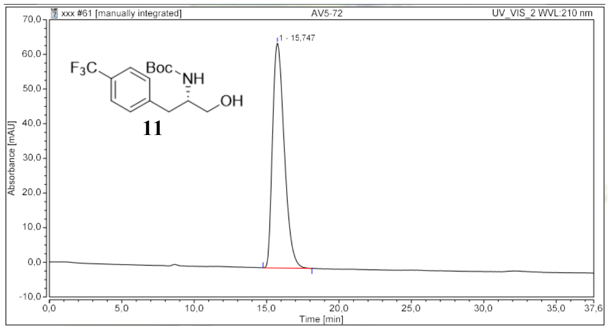

HPLC chromatograms for 16l (TUG-2304)

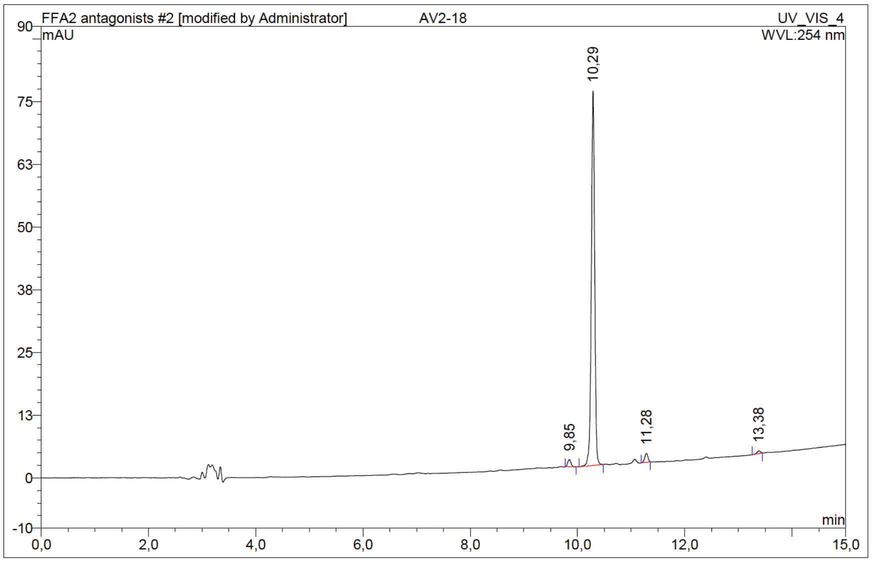

No.	Ret.Time min	Peak Name	Height mAU	Area mAU*min	Rel.Area %	Amount	Resolution(EP)
1	9,85	n.a.	1,423	0,096	1,67	n.a.	3,99
2	10,29	n.a.	74,642	5,506	95,57	n.a.	9,09
3	11,28	n.a.	1,762	0,119	2,07	n.a.	18,33
4	13,38	n.a.	0,526	0,040	0,69	n.a.	n.a.
Total:			78,353	5,761	100,00	0,000	

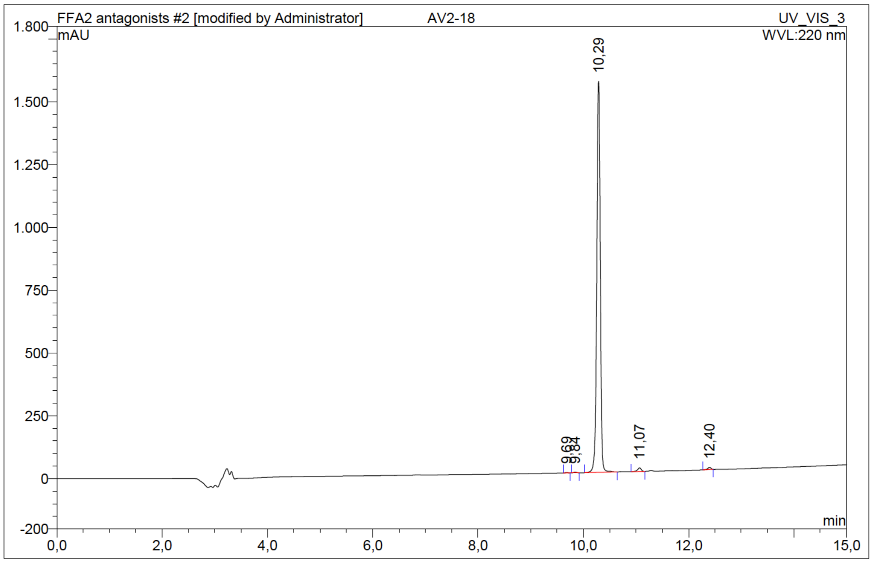

No.	Ret.Time min	Peak Name	Height mAU	Area mAU*min	Rel.Area %	Amount	Resolution(EP)
1	9,69	n.a.	1,385	0,093	0,08	n.a.	1,43
2	9,84	n.a.	2,877	0,190	0,16	n.a.	4,05
3	10,29	n.a.	1555,627	115,970	98,21	n.a.	6,55
4	11,07	n.a.	14,746	1,218	1,03	n.a.	11,28
5	12,40	n.a.	8,432	0,608	0,51	n.a.	n.a.
Total:			1583,068	118,079	100,00	0,000	

HPLC chromatograms for 16w

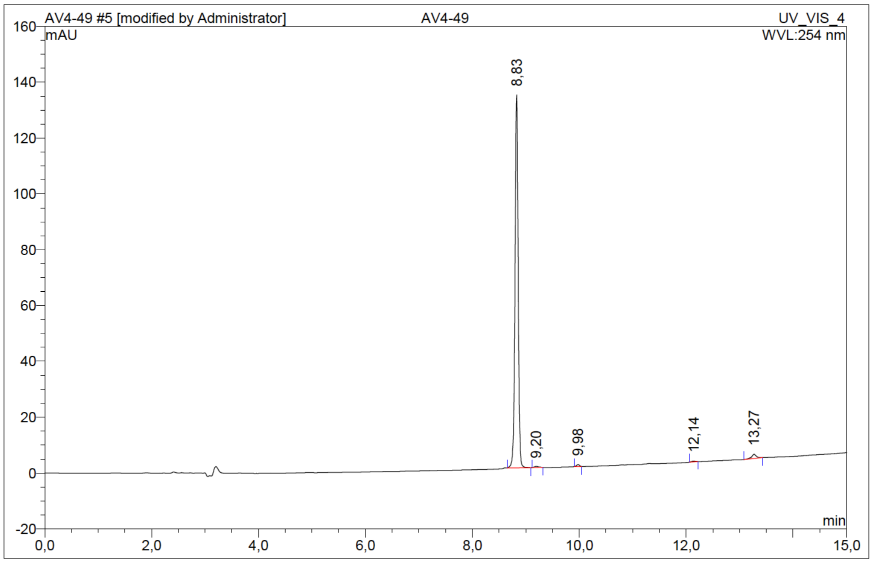

No.	Ret.Time min	Peak Name	Height mAU	Area mAU*min	Rel.Area %	Amount	Resolution(EP)
1	8,83	n.a.	133,610	9,279	97,40	n.a.	2,82
2	9,20	n.a.	0,368	0,035	0,37	n.a.	6,10
3	9,98	n.a.	0,693	0,043	0,45	n.a.	17,06
4	12,14	n.a.	0,245	0,022	0,23	n.a.	7,81
5	13,27	n.a.	1,479	0,147	1,54	n.a.	n.a.
Total:			136,396	9,527	100,00	0,000	

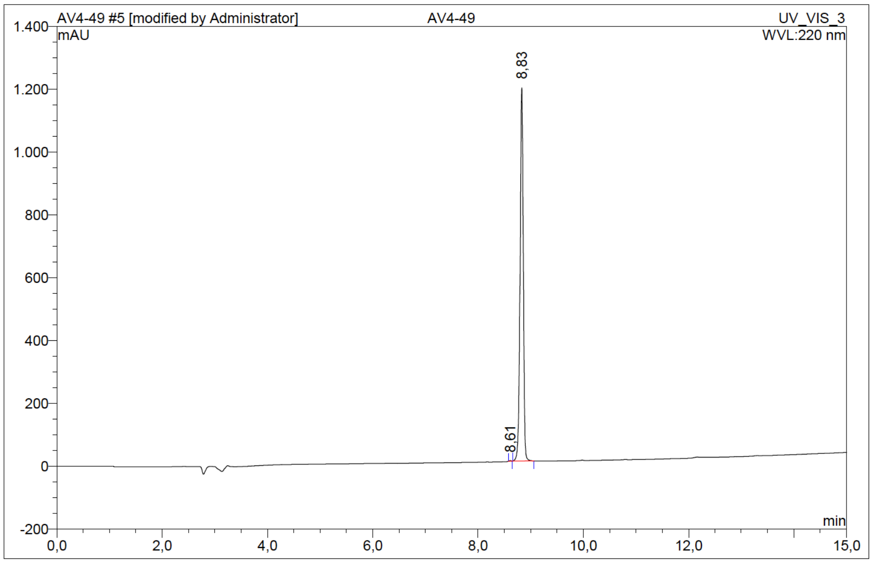

No.	Ret.Time min	Peak Name	Height mAU	Area mAU*min	Rel.Area %	Amount	Resolution(EP)
1	8,61	n.a.	1,379	0,056	0,07	n.a.	2,43
2	8,83	n.a.	1187,622	83,220	99,93	n.a.	n.a.
Total:			1189,000	83,276	100,00	0,000	

References

(1) Parmeggiani, F.; Lovelock, S. L.; Weise, N. J.; Ahmed, S. T.; Turner, N. J. Synthesis of D- and L-phenylalanine derivatives by phenylalanine ammonia lyases: a multienzymatic cascade process. *Angew. Chem. Int. Ed.* **2015**, *54*, 4608-4611.
